# Phosphoramidate Derivatives of Betulin, New Molecules with Promising Biological Activity: Synthesis and Characterization

**DOI:** 10.3390/molecules31060935

**Published:** 2026-03-11

**Authors:** Elwira Chrobak, Marta Świtalska, Marcel Madej, Joanna Wietrzyk, Ewa Bębenek

**Affiliations:** 1 Department of Organic Chemistry, Faculty of Pharmaceutical Sciences in Sosnowiec, Medical University of Silesia in Katowice, 4 Jagiellońska Str., 41-200 Sosnowiec, Poland; 2 Hirszfeld Institute of Immunology and Experimental Therapy, Polish Academy of Sciences, 12 Rudolfa Weigla Str., 53-114 Wrocław, Poland; marta.switalska@hirszfeld.pl (M.Ś.); joanna.wietrzyk@hirszfeld.pl (J.W.); 3 Department of Molecular Biology, Faculty of Pharmaceutical Sciences in Sosnowiec, Medical University of Silesia, 40-055 Katowice, Poland; marcel.madej@sum.edu.pl; 4 Silesia LabMed, Centre for Research and Implementation, Medical University of Silesia in Katowice, 18 Medykow Str., 40-752 Katowice, Poland

**Keywords:** betulin, phosphoramidate, anticancer activity, derivatives, apoptosis, caspases

## Abstract

Studies of natural products and their semisynthetic derivatives are a valuable source of therapeutic agents. The aim of this work was to obtain new 30-phosphoramidate derivatives of betulin and determine their biological potential. The synthetic approach utilized the Staudinger reaction (the introduction of a phosphoramidate group), the Steglich reaction (the introduction of an alkynyl group), and the Jones reaction (the introduction of a carboxyl group). The structures of the target compounds were determined using spectroscopic methods (^1^H NMR, ^13^C NMR, ^31^P NMR, and HRMS). The new derivatives were tested for antiproliferative activity against MV4-11, A549, MCF-7, PC-3, and HCT116 cancer cells and against normal MCF-10A cells using the MTT and SRB methods. Apoptosis studies were performed for the most active compounds (**6B** and **7A**), potential molecular targets (AutoDock software) were identified, and lipophilicity parameters (RP-TLC method, SwissADME website) were determined. The greatest effect on apoptosis and caspase 3/7 activation was observed for the diester derivative **7A**. Compound **7A** showed a high lipophilicity parameter in the study group.

## 1. Introduction

Cancer is one of the most difficult health problems to treat. This is due to the complex changes that underlie it, often developing over many years, as well as the low selectivity, high toxicity, and evolving resistance mechanisms of the chemotherapy agents used. In recent years, numerous discoveries have been made in understanding cancer processes and their mechanisms, which has also led to the significant development of new methods and directions in pharmacotherapy [1].

Among the most effective anticancer drugs currently in use, a large group are those derived from natural sources [2,3,4]. The search for new, effective anticancer drugs continues to rely on the use of natural products and their chemically modified derivatives. Natural compounds are also a rich source of leading structures and provide inspiration for the design of synthetic drugs used in new therapeutic approaches [5].

Betulin is a pentacyclic triterpene alcohol belonging to a series of lupane derivatives, which is obtained from the outer bark of birch trees. Betulin and its oxidized form, betulinic acid, exhibit multifaceted biological activity, including anticancer, antiviral, antimicrobial, antiallergic, and hepatoprotective effects [6,7,8]. Due to their low toxicity, demonstrated in both in vitro and in vivo studies, these compounds are of interest to scientists. The main limitation to the use of betulin as a therapeutic agent is its poor solubility in body fluids and insufficient intracellular accumulation. The in vivo bioavailability of betulin can be improved by modifying its native structure, which improves solubility in water and, therefore, in body fluids [9,10]. Numerous studies are being conducted involving the chemical transformation of these compounds to obtain derivatives with higher activity and better bioavailability [11].

The applied structural changes mainly focused on the functionalization of alcohol groups at the C-3 and C-28 positions in betulin or the acid group at the C-17 position in betulinic acid, which led to the formation of esters, amides, carbamates, sulfates, and phosphates [12,13,14,15]. Another direction of chemical transformations of betulin and betulinic acid is the synthesis of derivatives modified at the C-30 position of the triterpene skeleton. The introduction of new functions at this position results in compounds with anticancer (esters, oxo-amides, 1,2,4-triazoles, *N*-acylhrazones), antiviral (phosphonates), antibacterial (bromine), antifeedant (ester, epoxide), and antischistosomal activity (triphenylphosphonium) [10,16,17,18,19,20,21,22,23]. Functionalization of lupanes at the isopropenyl substituent (C-29 and C-30 positions) also involved the introduction of phosphorus-containing groups such as phosphate, phosphonate or triphenylphosphonium moieties [23,24,25,26].

Phosphorus compounds have their place in medicinal chemistry. In pharmacotherapy, they phosphonate derivatives are used as antibiotics (fosfomycin), antivirals (foscarnet, cidofovir, adefovir, pradefovir), antifungals (rhizocticin), antimicrobials (plumbemycin), and antimalarials (fosmidomycin) [27,28]. A phosphate group is often present in prodrugs, improving the bioavailability and pharmacological activity of the compounds. Examples include the antiviral fosamprenavir (an amprenavir derivative). Fosphenytoin is used to control seizures, fosfluconazole is used for fungal infections, fludarabine phosphate, a purine nucleoside antitumor agent, and estramustine for the treatment of advanced prostate cancer, and fospropofol is used for sedation in diagnostic or medical procedures. Fostamatinib disodium has been approved for use in rheumatoid arthritis and immune thrombocytopenic purpura, while tedizolid phosphate is used for bacterial infections of the skin and subcutaneous tissue [27].

**Figure 1:**
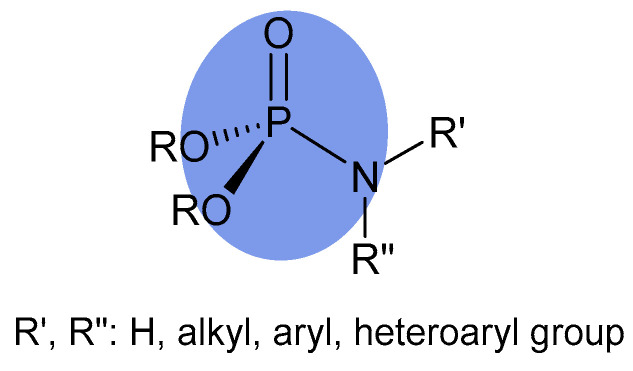
General formula of phosphoramidate.

A separate group consists of derivatives with a phosphoramidate group, in which, in addition to the single P-O and phosphoryl group (P=O), the P(V) atom is connected by a single covalent bond to the N(III) atom (Figure 1) [29].

Since the 1940s, chemists have developed numerous methods for the formation of a phosphoramidate group. Among these, the most commonly used methods have been the reaction of azides with phosphites (Staudinger phosphite reaction), the coupling reaction between an amine and a dialkyl dialkylphosphite (Atherton-Todd reaction), amines with phosphoryl halides, iodine-mediated phosphoramidation of amines, and the oxidative coupling of amines and H-phosphonates in the presence of Cu(I) [29,30].

Compounds with phosphoramidate structures exhibit a broad range of biological activities, including anticancer, antioxidant, anti-microbial, antimalarial, and antiviral effects, such as anti-HIV activity [31,32,33,34,35,36]. In nucleotide chemistry, the phosphoramidate group occurs as a building block in the synthesis of phosphorus esters [37].

It is also worth mentioning the FDA-approved drug sofosbuvir, which is used for the treatment of hepatitis C virus (HCV). In recent years, remdesivir, another drug also containing a phosphoramidate moiety, has been evaluated as a therapeutic option for patients with COVID-19 [38,39].

In the literature on chemically modified derivatives of pentacyclic triterpenes, only one paper can be found on phosphoramidate derivatives of betulinic acid. The compounds obtained by the authors were tested on human cell lines: breast cancer (MCF-7), lung cancer (A549), colon cancer (HCT-116), leukemia (MOLT-4), prostate cancer (PC-3), and pancreatic cancer (MiaPaca-2) [40].

On the other hand, the carbon–carbon triple bond has already established a strong position in medicinal chemistry as a moiety with potential biological activity. This is supported by a substantial number of approved drugs, as well as numerous scientific papers reporting promising results for novel chemical structures containing this substituent [41,42].

**Figure 2:**
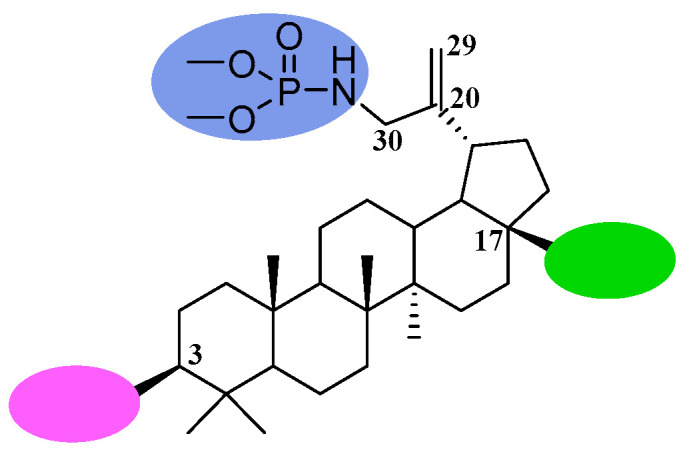
General structure and positions of modifications of the betulin skeleton for obtaining the title compounds.

Considering the above information, it seemed interesting to test whether the strategy of combining the phosphoramidate and alkynyl functions in a single molecule would prove beneficial for anticancer activity. Therefore, we planned the synthesis of 30-phosphoramidate derivatives of betulinic acid as well as 30-phosphoramidate derivatives of 28- or 3- and 28-alkynylbetulins shown in Figure 2.

To obtain betulin derivatives with a phosphoramidate group, the Staudinger method was chosen in this study, which utilizes the reaction of an azide group with a trialkyl phosphite [40,43]. This method was used for the modification of betulin due to the ease of obtaining starting materials, mild reaction conditions, and simple isolation of the final product.

The resulting derivatives were tested for their antiproliferative activity against selected cancer cell lines and their effect on apoptosis. Initial characterization of the new compounds included an assessment of their lipophilicity using reversed-phase thin-layer chromatography (RP-TLC).

## 2. Results and Discussion

### 2.1. Chemistry

**Figure 3:**
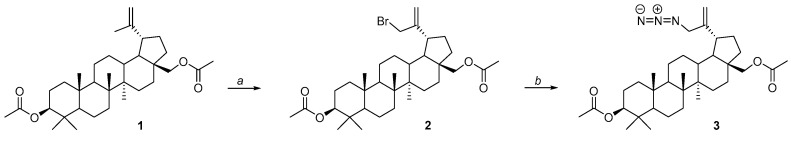
Synthesis scheme for 3,28-di-*O*-acetyl-30-azidobetulin **3**. Reagents and conditions: *a*—NBS, 1,4-dioxane, ambient temperature, overnight; *b*—NaN_3_, DMF, 100 °C, 12 h.

For the synthesis of the title betulin derivatives, 3,28-diacetylbetulin **1** was used, which was converted to the 30-bromosubstituted derivative **2** (Figure 3 step *a*), and then, by reaction with sodium azide, 3,28-di-*O*-acetyl-30-azidobetulin **3** was obtained (Figure 3 step *b*).

The synthesis of compound **3** was previously described by Uzenkova and Antimonova, and its product was used as a substrate in the synthesis of betulin derivatives containing a triazole ring or amino groups in the 30-position [44,45].

**Figure 4:**
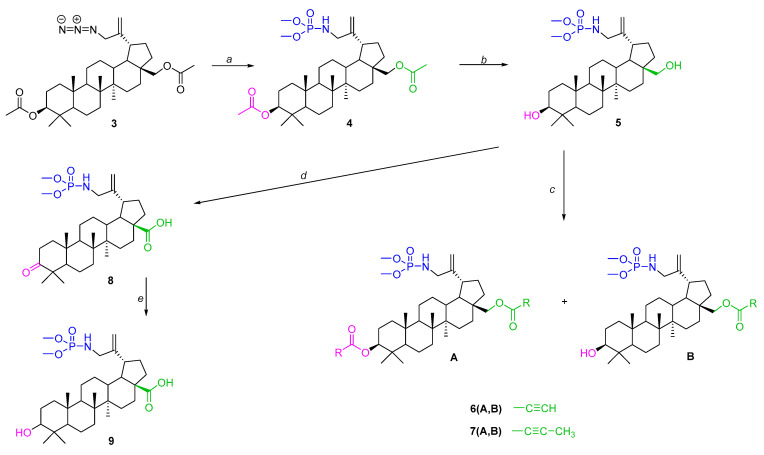
Synthesis scheme for the title derivatives of betulin **4, 5**, **6A**, **6B**, **7A**, **7B, 8** and **9**. Reagents and conditions: *a*—(CH_3_O)_3_P, toluene, 40–45 °C, 18 h; *b*—NaOH, THF/CH_3_OH/H_2_O, ambient temperature, 7 h; *c*—carboxylic acid, DCC, DMAP, CH_2_Cl_2_, from −5 °C to room temperature, 29 h; *d*—CrO_3_, acetone, H_2_SO_4_, 1,5 h, 0–10 °C; *e*—NaBH_4_, THF, ambient temperature, 2 h.

In this work, compound **3** was reacted with trimethylphosphite, which allowed its conversion to 30-phosphoramidate of 3,28-diacetylbetulin **4** (Figure 4 step *a*). Alkaline hydrolysis of this derivative yielded compound **5** with free hydroxyl groups at the 3 and 28 positions (Figure 4 step *b*). The resulting basic structure **5** was transformed into two types of derivatives, one of which were an alkynyl acid ester (**6A**, **6B**, **7A**, **7B**) of betulin phosphoramidate (Figure 4 step *c*), and the other was an oxidation product (phosphoramidate derivatives of betulonic acid **8** and betulinic acid **9**) (Figure 4 step *d*).

The structures of the compounds were determined by nuclear magnetic resonance spectroscopy (^1^HNMR, ^13^CNMR, ^31^PNMR) and HRMS mass spectrometry. NMR spectra of the target compounds were recorded in CDCl_3_.

An important element of the spectroscopic analysis was the confirmation of the introduction of a phosphoramidate substituent at carbon 30. First, the ^1^H NMR spectra of compounds **3** and **4** showed the absence of a signal from the three protons of the methyl group, which in the unsubstituted betulin system is visible as a singlet at δ = 1.7 ppm. Instead, a multiplet signal from the two protons of the CH_2_ group (in the substituted methyl group) is visible for compound **3** at δ = 3.78 ppm and for compound **4** at 3.48 ppm. To confirm the presence of a phosphorus atom in the molecules of the new compounds, ^31^P NMR spectra of the obtained derivatives were obtained. The chemical shift values for compounds **4**, **5**, **6A**, **6B**, **7A**, **7B**, **8** and **9** were in the range δ 11.10–11.71 ppm. Similar shift values (δ = 11.58 ppm) of the phosphorus signal in the dimethylphosphoramidate group were also reported by other authors [46].

### 2.2. Antiproliferative Activity Study

The compounds described so far, containing phosphorus substituents in the isopropenyl fragment of the betulin skeleton, have been studied as antiviral agents (HIV-1; Human Immunodeficiency Virus, BEV, ECBO; cytopathogenic bovine orphan virus, VSV; vesicular stomatitis virus, HHV-1; herpes simplex virus type I and AHAdV-5; human adenovirus type-5) [18,26]. Anticancer activity studies have been described for 30-diethylphosphate and 29-diethylphosphonate derivatives of alkynylbetulin and 30-triphenylphosphonium analogues of betulin and betulinic acid additionally containing ester or amide groups in positions 28 or 3 and 28 [23,24,25].

The antiproliferative activity of the title betulin derivatives **4, 5**, **6A**, **6B**, **7A**, **7B, 8** and **9** was assessed. The human cell lines selected for this study included biphenotypic myelomonocytic leukemia (MV4-11), as well as breast (MCF-7), lung (A549), prostate (PC-3), and colon (HCT116) tumor cell lines. The results, expressed as IC_50_ values (the inhibitory concentration at cell viability is reduced by 50%), are presented in Table 1.

Two reference compounds were used in this study: betulin as the basic system from which the remaining compounds were derived, and the anticancer drug doxorubicin. The IC_50_ values determined for betulin against the tested cancer cell lines ranged from 14.0 to 51.5, and in the case of the MV4-11 cell line, they were higher than for all of its derivatives. The exception was 30-phosphoramidate of betulin (compound **5**), which was not active against any of the tested cell lines, using a cut-off of 100 µM. Therefore, the introduction of a phosphoramidate group weakens the anticancer potential of betulin. Oxidation of the hydroxyl groups at positions 28 (the carboxyl group in compound **9**) or 3 and 28 (the carbonyl and carboxyl groups in compound **8**, respectively) leads to increased activity against the MV4-11 cell line. This pattern was not confirmed in the case of lung cancer (A549) and breast cancer cells (MCF-7).

Analyzing the antiproliferative activity of the tested group of ester derivatives of betulin phosphoramidate, a significant selectivity of the propynoyl diester **6A** can be observed. For this compound, an IC_50_ value of 0.092 µM was determined against leukemia cells, while it was inactive against the other cell lines. The monoester derivative **6B** exhibits promising antiproliferative activity against all tested cancer cells, with IC_50_ values ranging from 0.83 to 4.93 µM.

2-Butynoic acid esters containing a triple carbon–carbon bond are characterized by lower activity than propynoyl esters, in which this bond is located at the end of the substituent (terminal).

In order to determine the effect of the carboxyl (C-17) and hydroxyl (C-28) group on the antiproliferative activity of 30-phosphoramide derivatives of betulin, compounds **5** and **9** were synthesized. The presence of the carboxyl group in compound **9** causes an increase in activity against MV4-11, A549 and MCF-7 cells in comparison to derivative **5**. The obtained IC_50_ values for the phosphoramidate derivative of betulinic acid **9** in relation to A549 and MCF-7 cells are similar to the values determined earlier for this compound (for the A549 line: IC_50_ = 46.71 µM, for the MCF-7 line: IC_50_ = > 50 µM [40].

In studies evaluating the antiproliferative activity of new derivatives, it is essential to assess the selectivity of their action, as cytotoxic effects should be limited to non-cancerous cells. For selectivity analysis the normal human mammary epithelial cell line MCF-10A was used as a non-tumorigenic control. The selectivity index (SI) was determined by dividing the IC_50_ value for MCF-10A cells (Table 1) by the IC_50_ value for the specific tumor line under study. The SI values are presented in Table 2.

The general toxicity of the compound is when SI is less than 1.0. When SI is >1.0 it indicates the selective mechanism of action. The higher the SI value, the better the selectivity of the compound and the safer is for the organism. Derivatives **6B** and **7A** have a very high SI for MV4-11 leukemia cells (34 and 9.27, respectively) and PC-3 prostate cancer cells (7 and 7.57, respectively), greater than that calculated for betulin (3.01 and 1.91, respectively), while doxorubicin showed no selectivity compared to PC-3 cells (SI = 0.09).

In the conducted study, the most favorable activity, encompassing all cancer cell lines used, was observed for compounds **6B** and **7A**. Considering this, derivatives **6B** and **7A** were selected for further studies to determine their effect on apoptosis.

### 2.3. Determination of Apoptosis

Apoptosis, or programmed cell death, is a natural physiological process that allows the body to remove damaged, infected or unnecessary cells. The process of apoptosis involves two pathways, called the extrinsic and intrinsic pathways. Activation of the intrinsic pathway is associated with mitochondria. Loss of mitochondrial membrane integrity contributes to an apoptosome formation and activation of caspase-9, which then cleaves and activates procaspase-3/7. The extrinsic pathway is associated with receptors located in the plasma membrane. Induction of this pathway leads to activation of caspase-8, which cleaves the proapoptotic protein Bid and procaspase-3, thus contributing to the activation of the execution phase of the process [49].

**Figure 5:**
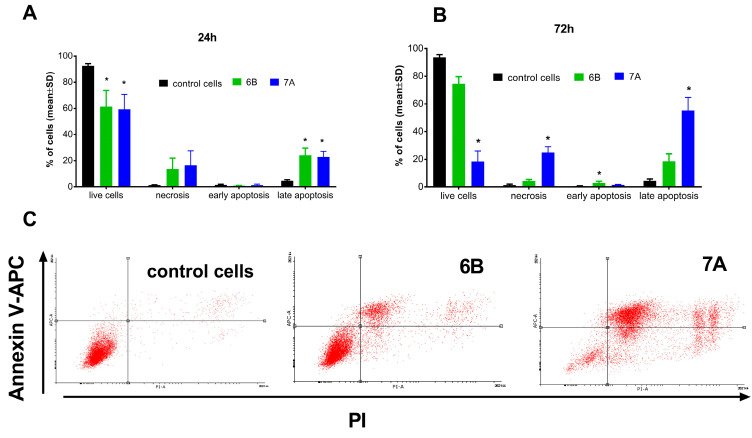
Cell death (apoptosis and necrosis) of PC-3 prostate cancer cells after 24 h (**A**) and 72 h (**B**,**C**) incubation with tested compounds. The **6B** compound in concentration of 1.7 µM and **7A** in concentration of 6 µM. (**A**,**B**)—mean with SD, (**C**)—dot plots from flow cytometry. * statistically significant versus control cells, *p* < 0.05, Kruskal–Wallis test.

To assess the effect of derivatives **6B** and **7A** on apoptosis, these compounds were added to cultured PC-3 prostate cancer cells at concentrations of approximately 2 × IC_50_ (1.7 and 6 µM, respectively). After 24 and 72 h of incubation, cells were stained with propiodium iodide (PI) and APC-Annexin-V, and cell death (apoptosis and necrosis) was analyzed by flow cytometry. After shorter time of incubation (24 h) induction of prostate cancer cells apoptosis (about 23% of cells, AV+/PI+) and necrosis (about 13% of cells, AV−/PI+) was observed (Figure 5A).

Prolonged incubation (72 h) of PC-3 cells with compound **6B** did not affect its ability to induce cell death. In contrast, after 72 h, treatment with compound **7A** resulted in a significant increase in PC-3 cell death (Figure 5B,C). Approximately 55% of the cells were apoptotic (AV+/PI−), about 25% were necrotic (AV−/PI+), and only 18.5% remained viable (AV−/PI−), compared with 93% viable cells in the control group.

### 2.4. Caspase-3/7 Activity Determination

Studies of the mechanism of action of triterpenoids on various cancer cells have shown that they can induce apoptosis by activating the caspase signaling pathway. Sequential activation of caspases plays an important role in cell apoptosis, and caspase-3 is a key executor of apoptosis [50,51,52,53].

**Figure 6:**
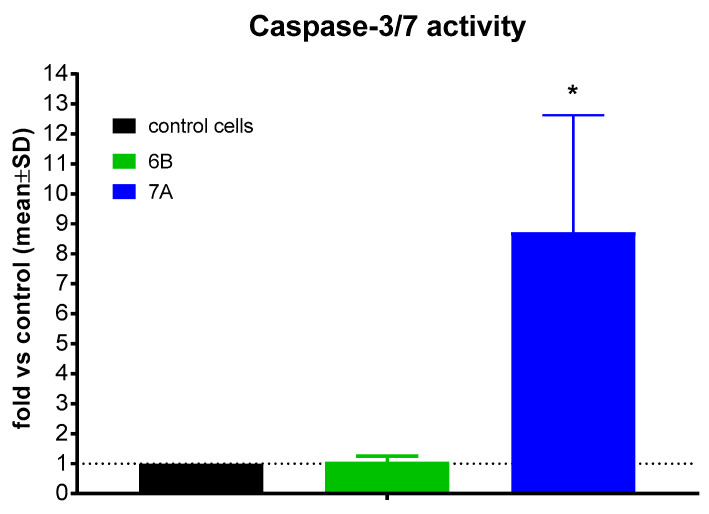
Caspase-3/7 activity of compounds **6B** and **7A** after 72 h. * statistically significant versus. control, *p* < 0.05.

To elucidate the mechanism of apoptosis induction by the tested compounds, their effect on caspase-3/7 activity was evaluated in PC-3 cells. Following 72 h treatment with compounds **6B** and **7A** at concentrations of 2 × IC_50_ (1.7 µM and 6 µM, respectively), caspase-3/7 activity was assessed. No caspase-3/7 activation was detected after exposure to compound **6B**, whereas treatment with compound **7A** resulted in a marked increase in caspase-3/7 activity, approximately 8.5-fold higher than in control cells (Figure 6).

Based on our results, we can only conclude that compound **6B** induces cell death (apoptosis and necrosis) without caspase-3 activation. Therefore, cell death likely occurs via a caspase-independent pathway, in contrast to compound **7A**, which induces caspase-3-dependent cell apoptosis. Determining which type of caspase-independent cell death (e.g., autophagy, mitotic catastrophe, or necroptosis) is induced by compound **6B** would require very detailed studies, going beyond the scope of preliminary analyses aimed at selecting the most favorable chemical modification of betulin.

### 2.5. Molecular Docking Analysis of Selected Compounds Targeting FLT3 and CASP3 Proteins

MV4-11 leukemia cells and PC-3 prostate cancer cells were found to be the most sensitive to the tested compounds (Table 1). MV4-11 cells are used in studies of acute myeloid leukemia (AML). They are characterized by various genetic abnormalities, including the expression of FLT3 receptors with an ITD mutation, which is useful for establishing an in vitro model for studying the disease and experimentally evaluating inhibitors [54,55]. The in vitro cytotoxic activity induced in the PC-3 prostate cancer cell line was found to be associated with caspase-3 modulation. This result was confirmed for compound **7A**, which increases its activity.

To elucidate the molecular basis of action of the synthesized derivatives and to assess their potential interaction strength with the investigated cells, molecular docking studies were conducted against the FLT3 and CASP3 proteins.

To perform molecular docking to determine the potential of the tested compounds as potential tyrosine kinase inhibitors, the crystal structure of FLT3 (PDB ID: 6JQR) was selected. Binding energy (ΔG) values for the analyzed compounds are listed in Table 3 (lower ΔG values correspond to increased ligand–protein affinity).

Among the tested compounds, **6A** and **6B** showed the most favorable binding profiles toward FLT3, as evidenced by their lowest binding energy values (Table 3).

**Figure 7:**
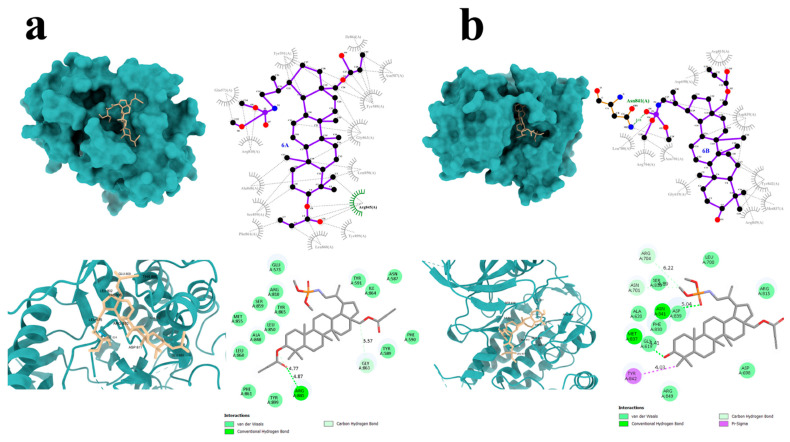
Graphical interaction of FLT3 (PDB ID: 6JQR) protein with the selected compounds: **6A** (**a**) and **6B** (**b**); green color indicates hydrogen bonds.

Detailed visualization of the docking poses was performed to characterize the interactions of compounds **6A** and **6B** with the FLT3 protein (Figure 7). Compound **6A** formed a single hydrogen bond with the Arg845 residue of the target protein (Figure 7a), along with four carbon–hydrogen bonds and several additional hydrogen-bonding interactions.

Compound **6B**, which displayed more favorable binding energy parameters, established two hydrogen bonds with the amino acid residues Asn841 and Met837 (Figure 7b). Notably, π–sigma interactions involving the Tyr842 residue were also observed in the FLT3–6B complex. Similar to compound **6A**, compound **6B** formed multiple carbon–hydrogen and conventional hydrogen bonds.

DOX, used as the reference compound, formed two hydrogen bonds with the amino acid residues Arg849 and Arg810. Additionally, carbon–hydrogen interactions were observed with Glu573 and Tyr572. Numerous van der Waals and alkyl interactions were also present, further stabilizing the complex.

To assess the effects of the synthesized derivatives on CASP3, a key protein involved in apoptosis, molecular docking studies were also performed [56,57]. Among the tested compounds, derivatives **6B** and **7A** exhibited the most favorable binding energy values, which were comparable to or better than those of the reference compound, doxorubicin (Table 4).

**Figure 8:**
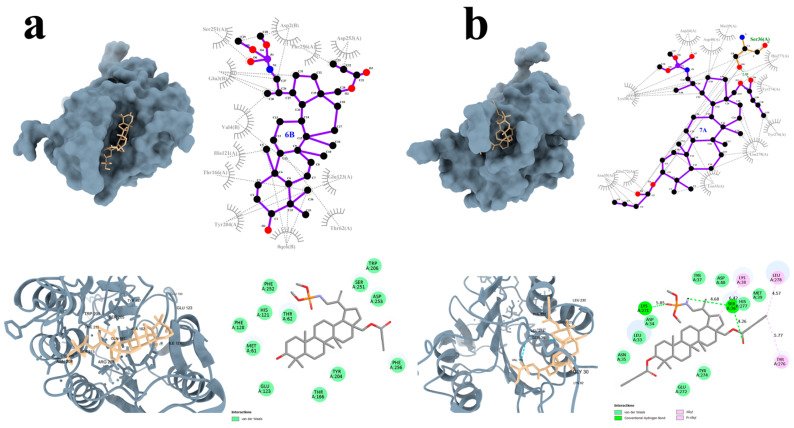
Graphical interaction of CASP3 (PDB ID: 5I9B) protein with the selected compounds: **6B** (**a**), **7A** (**b**); green color indicates hydrogen bonds.

Selected derivatives **6B** and **7A** were further visualized to gain a detailed understanding of their interactions with CASP3 (Figure 8). Interestingly, compound **6B** did not form any hydrogen bonds however established numerous van der Waals interactions (Figure 8a). Notably, the structure of **6B** is additionally positioned within a larger protein pocket, which may contribute to its strong binding affinity.

In contrast, compound **7A** formed two hydrogen bonds with the CASP3 residues Ser26 and Lys271. Additionally, π–alkyl and alkyl interactions were observed with the amino acid residues Lys38, Tyr276, and Leu278. Multiple van der Waals interactions were also present, further stabilizing the compound within the binding site (Figure 8b).

DOX formed a hydrogen bond with the amino acid residue Thr174, as well as carbon–hydrogen interactions with Thr245 and alkyl interactions with Arg241. In addition, numerous van der Waals contacts were observed, contributing to the stabilization of the complex.

Considering the experimental and computational results, it can be observed that the interaction of compounds **6B** and **7A** with selected proteins (for the MV-4-11 line—FMS-like Tyrosine Kinase 3/FLT3 and for the PC-3 line—caspase3) may suggest potential molecular mechanisms responsible for their effects on the studied leukemia and prostate cancer lines.

### 2.6. Lipophilicity Study

In the early stages of developing new bioactive molecules, in vitro studies are crucial, as are in silico analyses, which are becoming increasingly important because they can be performed more quickly and cost-effectively [58]. In addition to confirming the specific biological activity of a new compound, it is necessary to understand its physicochemical properties and the potential activities it may exhibit in the biological environment. Among numerous molecular descriptors, lipophilicity plays a significant role as the most important physicochemical parameter of a molecule. It significantly influences bioactivity and largely determines the transport of substances across the cell membrane, influencing the formation of complexes between the tested substances and plasma proteins or the appropriate receptor at the site of action [59,60,61].

RP-TLC is a convenient method for testing lipophilicity due to its simplicity and low instrumentation requirements, the use of small amounts of the substance, accuracy, reproducibility, and efficiency, as well as the ability to differentiate lipophilicity between structurally similar molecules. The advantage of this method is the ability to simultaneously analyze a large number of samples [62,63].

The RP-TLC method involves the use of two phases: stationary and mobile. The nonpolar and hydrophobic stationary phase is silica gel modified by the addition of long carbon chains (e.g., C8, C18). The most commonly used is octadecylsilane gel (RP-18), with 18 carbon atoms. The mobile phase is a polar solvent system, most often an aqueous solution of acetone or methanol, but other solvents such as ethanol, tetrahydrofuran, dioxane, or acetonitrile are also used [64].

Retention in reversed-phase thin-layer chromatography depends primarily on the ability of the substance to retain on the stationary phase during migration with the mobile phase. The lipophilicity descriptor calculated from RP-TLC measurements is expressed by the equation:

R_M_ = log [(1/R_f_) − 1]

where R_f_ is the retention coefficient, determined from the chromatograms of the tested substances by measuring the distance traveled by the tested sample (L_sample_) and the distance traveled by the solvent (L_solv_).

R_f_ = L_sample/_L_solv_

The normalized chromatographic lipophilicity parameter R_M0_ is determined based on the dependence of the R_M_ parameter on the volume composition of the mobile phase, i.e., the proportion of the organic component. This dependence is linear and is expressed by the following formula:

R_M_ = R_M0_ + bC

where the following definitions are used:

C—concentration of the organic phase—volume fraction of the organic component in the mobile phase;

b—slope of the linear dependence.

The R_M0_ parameter is obtained by extrapolation to zero concentration of the organic component [65].

R_M0_ = R_M_ − bC

The R_M0_ values obtained from the chromatographic experiment can be used to determine the logP_TLC_ lipophilicity parameter. This requires the use of a set of appropriately selected standard substances with a known logP_lit_ value (Supplementary Materials) covering the lipophilicity range of the tested compounds to determine a calibration curve, which is the relationship between logP_lit_ and R_M0_ (for the standards used). From the equation of this curve (Supplementary Materials), using the previously calculated R_M0_ values for the tested samples, we read the logP_TLC_ values (Table 5).

The lowest logP_TLC_ values were determined for derivatives containing polar groups at the 3-position, such as hydroxyl, carbonyl, and carboxyl groups (**5**, **8**, and **9**). Conversion of the hydroxyl group at the C-28 position into an ester function with a triple bond in the substituent increases lipophilicity (compounds **6B** and **7B**). The additional introduction of the same ester group at the C-3 position results in a further increase in lipophilicity (compounds **6A** and **7A**). Depending on the structure of the substituent in the ester group, the lipophilicity of disubstituted derivatives increases in the following order: **4** (acetyl) < **7A** (butynoyl) < **6A** (propynoyl). Considering compounds (**6B** and **7A**) that exhibit the highest in vitro antiproliferative activity against MV4-11 and PC-3 cells, it can be seen that higher activity is associated with lower logP_TLC_ lipophilicity values.

Increased lipophilicity of compounds is commonly associated with enhanced penetration through cellular membranes, potentially contributing to membrane-targeting activity [66,67]. Based on this, compounds **6B** and **7A** are likely to exert their effects through distinct membrane-related mechanisms. Compound **6B**, despite its lower lipophilicity than compound **7A**, demonstrates higher cytotoxicity and does not activate caspases 3 and 7. This behavior may result from its tendency to aggregate at cellular membranes, causing destabilization and inducing apoptosis-independent cell death. At lower concentrations, it may directly compromise cell viability by disrupting membrane structure. Conversely, compound **7A** exhibits higher lipophilicity, which may facilitate deeper insertion into cellular membranes. Consequently, it may trigger cell death via a caspase 3- and 7-dependent pathway, consistent with the observed increase in enzymatic activity in treated cells. These differences in lipophilicity likely underpin the distinct membrane effects and modes of cell death observed—apoptosis-independent for **6B** and caspase-dependent for **7A**. Nonetheless, further studies are needed to confirm the proposed membrane-targeting mechanisms, and the current description remains a hypothetical interpretation of the observed effects. Similar findings were reported by Belosludtsev et al., who investigated the BA-F16 conjugate in breast cancer cells and normal fibroblasts. They observed that introducing the lipophilic F16 cation into betulinic acid enhanced both cytotoxic and membrane-targeting effects by increasing lipid membrane permeability [68].

Based on five computational models (iLogP, MLOGP, WLOGP, SILICOS-IT, and XLOGP3), theoretical logP values were determined for the tested compounds and are presented in Table 6. The lowest logP values were obtained from the MLOGP and iLogP programs, while the highest were obtained from XLOGP3. The experimentally determined logP_TLC_ values were compared with the theoretical logP values. It was observed that compounds **5**, **8**, and **9** were characterized by the lowest values of both the logP_TLC_ parameter (logP_TLC_ = 3.97–5.00) and the average value of the calculated logP parameter. A similar relationship occurs for derivative **6A**, for which the highest value of the lipophilicity parameter was determined experimentally and theoretically.

Analysis of the results obtained from the theoretical programs indicates that the highest correlation coefficient (R = 0.989) occurs between the MLOGP and XLOGP3 programs. For the experimental values of logP_TLC_, the highest correlation coefficient was obtained after comparison with the theoretical values of iLogP and MLOGP (R = 0.856–0.857, Table 7).

## 3. Materials and Methods

Reagents and solvents used in the synthesis were purchased from Merck (Darmstadt, Germany). The structures of the new compounds were confirmed by melting point measurements and spectroscopic analysis (^1^H, ^13^C, ^31^P NMR). The melting points (mp) of the new betulin derivatives were determined using an Electrothermal IA 9300 instrument (Electrothermal, Rochford, UK). ^1^H, ^13^C, and ^31^P NMR spectra (600 MHz, 150 MHz, and 243 MHz, respectively) were obtained from samples prepared by dissolving the compounds (10 mg) in deuterated chloroform (CDCl_3_) on a Bruker Avance III 600 instrument (Bruker Corporation, Billerica, MA, USA). HRMS spectra were obtained on a Bruker Impact II instrument (Bruker Corporation, Billerica, MA, USA) in negative ion mode (APCI). Silica gel 60 254F TLC plates (Merck, Darmstadt, Germany) were used to monitor the reaction. Mixtures of hexane with ethyl acetate (8:1) and dichloromethane with ethanol (15:1, 30:1, and 40:1) were used as eluents. The obtained chromatograms were visualized by spraying with 10% ethanolic H_2_SO_4_ and then heating to 100 °C. Column chromatography [solid phase silica gel 60 (0.063–0.200 mm, Merck, Darmstadt, Germany)] and an appropriate mobile phase were used for purification of betulin derivatives.

### 3.1. Experimental Procedures—Chemistry

**Figure 9:**
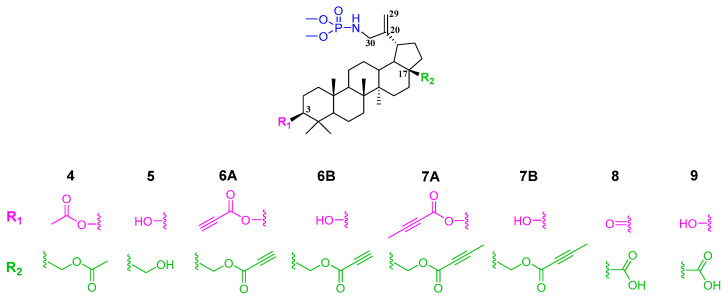
General structure of the phosphoramidate derivatives synthesized in this work.

As part of the work, 28 or 3 and 28 substituted derivatives of betulin 30-phosphoramidate were synthesized, the general structure of which is shown in Figure 9.

### 3.2. Procedure for the Synthesis of Target Compounds

In the first step, 3,28-diacetylbetulin **1** was brominated with *N*-bromosuccinimide at the allyl position (carbon atom at C-30 position, isopropenyl group) according to the previously described procedure [44]. The 3,28-diacetyl-30-bromobetulin **2** thus obtained was transformed into 3,28-di-*O*-acetyl-30-azidobetulin **3**, which became the starting material for the syntheses that are the subject of this work. The determined melting points and the results of spectroscopic analyses of compounds **2** and **3** were consistent with the literature information [44,45].

#### 3.2.1. Procedure for Preparation of 3,28-di-*O*-acetyl-30-azidobetulin **3**

A solution of 606 mg (1 mmol) of 3,28-diacetyl-30-bromobetulin **2** in 13 mL of DMF was placed in a reaction flask and 639 mg (1 mmol) of sodium azide (NaN_3_) was added. The reaction was carried out for 12 h at 100 °C. The reaction system was equipped with an air condenser and a calcium chloride tube to protect against moisture. The progress of the reaction was monitored by TLC (hexane/ethyl acetate, 8:1, *v*/*v*) until the substrate spot disappeared (R_fsubstr_ = 0.33; R_fprod_ = 0.27). After the reaction was completed, the mixture was diluted with 200 mL of ethyl acetate and then washed five times with 50 mL of water and once with 50 mL of brine. The organic layer was dried with anhydrous sodium sulfate. After separation of the drying agent (filtering), it was evaporated to dryness in a vacuum evaporator. The final product **3** was obtained (91% yield, 517 mg). The melting point was determined to be 190–191 °C. The literature value of the melting point was 192–193 °C.

#### 3.2.2. Reaction of Compound **3** with Trimethyl Phosphite by the Staudinger Method—Synthesis of Phosphoramidate 3,28-di-*O*-acetylbetulin **4**

In a flask protected against moisture (tube with CaCl_2_), a solution of 586 mg (1 mmol) of 3,28-di-*O*-acetyl-30-azidobetulin **3** in 15 mL of toluene was placed and then trimethyl phosphite (0.12 mL, 1 mmol) was added under an inert gas atmosphere (argon). The reaction was conducted on a magnetic stirrer at 40–45 °C. After 18 h, 20 mL of water was added and the reaction stirred vigorously for another 2 h at room temperature. The solution was then washed twice with 15 mL of water and once with 15 mL of brine. After separation, the organic layer was dried with anhydrous sodium sulfate, and the toluene was evaporated.

The obtained product **4** was purified by column chromatography in dichloromethane and ethanol (40:1, *v*/*v*) to obtain 518 mg of compound **4** (91.5% yield).

#### 3.2.3. Hydrolysis of the Acetyl Groups of Compound **4**—Synthesis of Compound **5**

A solution of sodium hydroxide in a mixture of methanol, THF, and water (1:2:1, *v*/*v*) was added to phosphoramidate of 3,28-diacetylbetulin **4**. The resulting solution was stirred at room temperature for 7 h, and then the volatile compounds were removed by rotary evaporation. 30 mL of dichloromethane was added to the residue. The organic layer was washed once with 5 mL of 10% hydrochloric acid, twice with 10 mL of water, and once with 10 mL of brine. After separation, the organic layer was dried with anhydrous sodium sulfate and concentrated to dryness using a rotary evaporator. 173 mg of methyl derivative **5** was obtained. The product was purified by column chromatography using dichloromethane and ethanol (15:1, *v*/*v*). 143 mg of derivative **5** was obtained (82% yield).

#### 3.2.4. Esterification of Compound **5** by the Steglich Method—Synthesis of Alkinyl Derivatives **6A**, **6B**, **7A** and **7B**

In a round-bottomed flask fitted with a calcium chloride tube to prevent moisture ingress, a solution of 0.4 mmol (240 mg) of betulin 30-phosphoramidate **5** in 2 mL of dichloromethane and 0.47 mmol of the appropriate acid was placed. The reaction vessel was placed in an ice-salt bath at −10 °C. Then, with magnetic stirring, a solution of 0.032 mmol (3 mg) of 4-(dimethylamino)-pyridine (DMAP) and 0.468 mmol (96 mg) of 1,3-dicyclohexylcarbodiimide (DCC) in 2 mL of dichloromethane was added dropwise. The reaction was continued under these conditions for 3 h and then gradually at room temperature. After 24 h, the precipitate was filtered off and washed twice with 5 mL of dichloromethane. The filtrate containing the mixture of expected compounds was concentrated to dryness in a vacuum evaporator. The obtained dry residues of crude products (**6A**,**B** −306 mg; **7A**,**B** −214 mg) were separated by column chromatography (silica gel Kieselgel 60, eluent: dichloromethane/ethanol, 15:1 *v*/*v*).

#### 3.2.5. Jones Oxidation Reaction of Compound **5**—Synthesis of the Phosphoramidate Derivative of Betulonic Acid **8**

To a solution of compound **5** (1 mmol) in distilled acetone (14 mL) cooled in an ice-water bath, we added 2.5 mL of Jones reagent (a mixture of chromium trioxide, water, and concentrated sulfuric acid). After stirring at room temperature for 1.5 h, the mixture was cooled, and 2.5 mL of ethanol was slowly added dropwise. Stirring was continued for another 15 min and then poured onto 40 g of finely crushed ice. The precipitate was filtered and, after drying, purified by column chromatography (Kieselgel 60 (<0.063%) in dichloromethane and ethanol (30:1, *v*/*v*). 1.005 g of compound **8** was obtained (32% yield).

#### 3.2.6. Reduction of the Carbonyl Group at the C-3 Position of Compound **8**—Synthesis of the Phosphoramide Derivative of Betulinic Acid **9**

To a solution of 525 mg (0.9 mmol) of compound **8** in 35 mL of methanol, we added 138 mg (3.6 mmol) of sodium borohydride (NaBH_4_) and stirred for 1.5 h. Then, 18 mL of 2.5% hydrochloric acid solution was added. The precipitate was dissolved in 20 mL of chloroform, separated, and the aqueous layer was extracted four times with 15 mL of chloroform. The chloroform extracts were concentrated, and the crude product was purified by column chromatography (chloroform/ethanol; 15:1, *v*/*v*) to give 399 mg of the product **9** (yield 76%).

Spectroscopic data for the obtained products are provided in the Supplementary Materials.

### 3.3. In Vitro Studies

#### 3.3.1. Biological Materials and Assays

All the human cell lines are maintained at the Hirszfeld Institute of Immunology and Experimental Therapy, Polish Academy of Sciences, Wrocław, Poland, and were obtained from the American Type Culture Collection (ATCC, Rockville, MD, USA) (leukemia MV4-11, colon cancer HCT116, normal epithelial cells from mammary gland MCF-10A) or from the European Collection of Authenticated Cell Cultures (Culture Collections UK Health Security Agency, Porton Down, Salisbury, UK) (lung cancer A549, breast cancer MCF-7 and prostate cancer PC-3). All cell lines were cultured in culture media described in detail previously [48] and were grown at 37 °C with 5% CO_2_ humidified atmosphere.

#### 3.3.2. Determination of Antiproliferative Activity

The tested compounds were dissolved in DMSO to a concentration of 20 mM and then were diluted in culture medium to reach the final concentrations. The cells were plated in 96-well plates at a density of 1 × 10^4^, 0.75 × 10^4^ (MCF-7) or 0.5 × 10^4^ (A549) cells per well (Greiner Bio One, Kremsmünster, Austria). After 24 h of incubation the tested compounds were added at the concentrations in the range of 100–0.02 µM. The cytotoxic effect of all agents was examined after 72 h of incubation by using the MTT (MV4-11) or SRB assay, described previously [48]. For each experiment (repeated 3–5 times) were calculated separately the values of IC_50_ (inhibitory concentration 50%) by using Prolab-3 system which based on Cheburator 0.4 software. Mean IC_50_ values with SD are listed in Table 1.

#### 3.3.3. Apoptosis Studies Using Annexin V and Propidium Iodide Staining

The human prostate PC-3 cells were seeded on 6-well plates (Greiner Bio One) and were exposed to compounds **6B** and **7A** at concentrations of about 2 × IC_50_ (1.7 μM and 6 μM, respectively) for 24 and 72 h. After incubation, the cells were collected, counted in hemocytometer and stained with APC-Annexin V (Becton Dickinson, Pharmingen, Franklin Lakes, NJ, USA) and propiodum iodide (PI, Sigma-Aldrich, Germany) in binding buffer according with procedure described previously [48]. Cell death was analysed by using flow cytometry in BD LSRFortessa cytometer (BD Bioscience, San Jose, CA, USA). Data were then analyzed using Flowing software 2.5.1 (Turku, Finland) and were displayed as a two-color dot plot with an APC-Annexin V vs. PI.

#### 3.3.4. Caspase-3/7 Activity Determination

The PC-3 cells were seeded on 24-well plates and were exposed to compounds **6B** and **7A** at concentrations of about 2 × IC_50_ (1.7 μM and 6 μM, respectively) for 72 h. After incubation the cultured medium was removed from the well and the cells were lysed in 50 µL of ice-cold lysis buffer (50 mM HEPES, 10% (*w*/*v*) sucrose, 150 mM NaCl, 2 mM EDTA, 1% (*v*/*v*) Triton X-100, pH 7.3, HIIET, Wrocław, Poland) and incubated 30 min at 4 °C. Then 40 µL of each sample was transferred to a white, 96-well plate (Corning, NY, USA) containing 160 µL of the reaction buffer (20 mM HEPES, 10% sucrose, 100 mM NaCl, 1 mM EDTA, 10 mM DTT, 0.02% Triton X-100, pH 7.3) (HIIET, Wrocław, Poland) with 9 µM AC-DEVD-AMC (Cayman Chemical, Ann Arbor, MI, USA) fluorogenic substrate (λ_ex_ = 360 nm, λ_em_ = 460 nm). Using a Biotek Synergy H4 plate reader (Biokom, Warsaw, Poland), the increase in fluorescence correlated with the caspase-3/7 level was continuously recorded for 120 min. at 37 °C. The experiment was repeated at least 3–4 times independently. Results were normalized to the number of cells in each well and are reported as mean relative caspase-3/7 activity compared to untreated control sample ± SD.

#### 3.3.5. Statistical Analysis

To indicate significant differences in the comparison of two groups (control vs. study), Kruskal–Wallis tests were performed using GraphPad Prism 7 software. A statistically significant difference was considered at *p* ≤ 0.05.

### 3.4. Procedure of Molecular Docking

To investigate potential interactions between the obtained compounds **4**, **6**, **6A**, **6B**, **7A**, **7B**, **8**, and **9** with the FLT3 protein, as well as compounds **4**, **6A**, **6B**, **7A**, and **7B** with CASP3, a molecular docking study was performed. This in silico approach was applied to elucidate possible mechanisms of action and to evaluate the binding affinity and selectivity of the tested ligands toward their respective target proteins [70].

The chemical structure of doxorubicin (DOX) was retrieved from the PubChem database [71] in SDF format and subsequently converted to MOL2 format using ChimeraX version 1.9. The chemical structures of the investigated compounds were generated by converting SMILES representations into three-dimensional PDB structures using the online NovoProLabs SMILES-to-PDB tool [72].

Crystal structures of the FLT3 (PDB ID: 6JQR) and CASP3 (PDB ID: 5I9B) proteins were obtained from the RCSB Protein Data Bank [73] in PDB format. Prior to docking, protein structures were prepared by removing crystallographic water molecules, co-crystallized ligands, and other non-essential components using ChimeraX 1.9. Further protein and ligand preparation was carried out in AutoDock Tools version 1.5.7, including the addition of polar hydrogen atoms and assignment of appropriate partial charges. The finalized protein and ligand structures were saved in PDBQT format for docking simulations. Redocking of the native ligand gilteritinib into the FLT3 crystal structure (PDB ID: 6JQR) was performed to validate the docking procedure, yielding an RMSD of 0.594 Å, confirming its accuracy.

Molecular docking calculations were conducted using AutoDock Vina version 1.1.2. The resulting for selected docking poses and protein–ligand interactions were analyzed and visualized using Discovery Studio 2025, ChimeraX 1.9, and LigPlot+ version 2.2.

## 4. Conclusions

In this work, a series of 30-phosphoramidate derivatives of betulin containing an alkynyl moiety at the C-3 and/or C-28 positions were synthesized. Activity tests were performed on five cancer cell lines and normal cells for the resulting compounds. For the most active phosphoamidates (**6B** and **7A**), which simultaneously exhibited the highest SI selectivity values, their effect on apoptosis in PC-3 cells was determined. Compound **7A** was selected as a caspase-3 activity enhancer. In the second part of the article, molecular docking of the synthesized compounds was performed to determine likely interactions with potential molecular targets (for the MV-4-11 line, FMS-like Tyrosine Kinase 3/FLT3, and for the PC-3 line, caspase 3). This study analyzed the antiproliferative activity and conducted a preliminary exploration of the mechanism of action (the effects of **6B** and **7A** derivatives on apoptosis), in conjunction with the results of lipophilicity testing of the new compounds. Based on this, it can be concluded that differences in lipophilicity (logP for **6B** and **7A** is 5.160 and 7.277, respectively) may be responsible for the different membrane effects and observed cell death modes (apoptosis-independent for **6B** and caspase-dependent for **7A**). The experimental results confirmed the beneficial effect of the alkynyl group in the phosphoramidate structure on activity.

## Figures and Tables

**Table 1:** Antiproliferative activity of the title compounds with betulin and doxorubicin as reference drugs.

Compound	IC_50_ [µM]					
	MV4-11	A549	MCF-7	PC-3	HCT116	MCF-10A
**4**	5.78 ± 2.1	10.1 ± 2.1	13.3 ± 1.5	12.37 ± 1.6	9.31 ± 0.9	22.4 ± 4.3
**5**	>100	>100	>100	nt	nt	nt
**6A**	0.092 ± 0.032	>100	>100	nt	nt	nt
**6B**	0.172 ± 0.022	2.65 ± 0.5	4.93 ± 0.9	0.83 ± 0.09	3.17 ± 0.36	5.86 ± 1.8
**7A**	2.46 ± 0.38	10.03 ± 2.0	17.67 ± 5.2	3.01 ± 0.9	13.69 ± 0.6	22.8 ± 0.8
**7B**	5.28 ± 1.4	9.44 ± 0.9	11.85 ± 0.3	11.76 ± 1.4	10.61 ± 1.2	12.05 ± 0.9
**8**	8.83 ± 3.8	35.5 ± 4.0	94.8 ± 2.8	nt	nt	nt
**9**	21.99 ± 1.8	37.5 ± 3.1	53.0 ± 2.7	nt	nt	nt
Betulin ^a^	32.75 ± 12.87	14.00 ± 1.13	43.59 ± 15.81	51.50 ± 9.94	37.72 ± 7.45	98.5 ± 0.9
Doxorubicin ^b^	0.021 ± 0.008	0.040 ± 0.012	0.150 ± 0.015	1.27 ± 0.2	0.118 ± 0.02	0.114 ± 0.013

(a) [47], (b) [48], (nt) no tested.

**Table 2:** Selectivity Index (SI) for derivatives **4**, **6B**, **7A** and **7B**.

Compound	Cell Lines/Calculated Selectivity Index SI				
	MV4-11	A549	MCF-7	PC-3	HCT116
**4**	3.88	2.22	1.68	1.81	2.4
**6B**	**34.07**	2.21	1.19	**7.06**	1.85
**7A**	**9.27**	2.27	1.29	**7.57**	1.67
**7B**	2.28	1.28	1.02	1.02	1.14
**Betulin**	3.01	7.04	2.26	1.91	2.61
**Doxorubicin**	5.43	2.85	0.76	0.09	0.97

The SI = IC_50_ for MCF-10A/IC_50_ for respective cancer cell line. A SI > 1.0 indicates a drug with efficacy against tumor cells greater than toxicity against normal cells.

**Table 3:** Binding affinities in complexes with FLT3 (PDB ID: 6JQR).

Compound	ΔG [kcal/mol]
**DOX**	−8.1
**4**	−5.6
**5**	−6.6
**6A**	−6.8
**6B**	−7.2
**7A**	−6.9
**7B**	−6.0
**8**	−5.7
**9**	−6.6

DOX—doxorubicin.

**Table 4:** Binding affinities in complexes with CASP3 (PDB ID: 5I9B).

Compound	ΔG [kcal/mol]
**DOX**	−7.7
**4**	−6.4
**6A**	−7.7
**6B**	−7.9
**7A**	−7.3
**7B**	−6.8

DOX—doxorubicin.

**Table 5:** The experimental values of lipophilicity parameters for target compounds.

Compound	R_M0_	r	*b*	logP_TLC_
**4**	5.69	0.988	−0.0647	7.003
**5**	3.04	0.981	−0.0389	3.971
**6A**	6.22	0.991	−0.0746	7.609
**6B**	4.08	0.986	−0.0502	5.160
**7A**	5.93	0.991	−0.0694	7.277
**7B**	4.23	0.983	−0.0513	5.332
**8**	3.61	0.985	−0.0478	4.623
**9**	3.94	0.982	−0.047	5.000

**Table 6:** The theoretical values of lipophilicity parameters for the tested compounds obtained from SwissADME [69].

Compound	iLogP	MLOGP	WLOGP	SILICOS-IT	XLOGP3
**4**	5.49	5.10	8.11	5.62	7.88
**5**	4.67	4.52	6.97	4.54	6.73
**6A**	6.09	5.28	7.50	6.13	8.83
**6B**	5.34	4.89	7.23	5.33	7.78
**7A**	5.71	5.63	8.28	6.67	9.86
**7B**	4.67	5.07	7.62	5.59	8.29
**8**	4.12	4.34	7.27	4.68	6.33
**9**	4.29	4.43	7.06	4.09	6.65

**Table 7:** Correlation matrix for theoretical and experimental lipophilicity data.

	iLogP	MLOGP	WLOGP	SILICOS-IT	XLOGP3	logP_TLC_
**iLogP**	1.000					
**MLOGP**	0.857	1.000				
**WLOGP**	0.594	0.830	1.000			
**SILICOS-IT**	0.841	0.969	0.824	1.000		
**XLOGP3**	0.821	0.989	0.777	0.959	1.000	
**logP_TLC_**	0.857	0.856	0.793	0.840	0.820	1.000

## Data Availability

The original contributions presented in the study are included in the article/Supplementary Materials; further inquiries can be directed to the corresponding authors.

## Supplementary Materials

The following supporting information can be downloaded at https://www.mdpi.com/article/10.3390/molecules31060935/s1: Spectroscopic data for compounds **3**–**5**, **6A**, **6B**, **7A**, **7B**, **8** and **9**. Figure S1. ^1^H NMR, compound **3**; Figure S2. ^13^C NMR, compound **3**; Figure S3. ^1^H NMR, compound **4**; Figure S4. ^13^C NMR, compound **4**; Figure S5. ^31^P NMR, compound **4**; Figure S6. HRMS, compound **4**; Figure S7. ^1^H NMR, compound **5**; Figure S8. ^13^C NMR, compound **5**; Figure S9. ^31^P NMR, compound **5**; Figure S10. HRMS, compound **5**; Figure S11. ^1^H NMR, compound **6A**; Figure S12. ^13^C NMR, compound **6A**; Figure S13. ^31^P NMR, compound **6A**; Figure S14. MS, compound **6A**; Figure S15. ^1^H NMR, compound **6B**; Figure S16. ^13^C NMR, compound **6B**; Figure S17. ^31^P NMR, compound **6B**; Figure S18. HRMS, compound **6B**; Figure S19. ^1^H NMR, compound **7A**; Figure S20. ^13^C NMR, compound **7A**; Figure S21. ^31^P NMR, compound **7A**; Figure S22. HRMS, compound **7A**; Figure S23. ^1^H NMR, compound **7B**; Figure S24. ^13^C NMR, compound **7B**; Figure S25. ^31^P NMR, compound **7B**; Figure S26. HRMS, compound **7B**; Figure S27. ^1^H NMR, compound **8**; Figure S28. ^13^C NMR, compound **8**; Figure S29. ^31^P NMR, compound **8**; Figure S30. HRMS, compound **8**; Figure S31. ^1^H NMR, compound **9**; Figure S32. ^13^C NMR, compound **9**; Figure S33. ^31^P NMR, compound **9**; Figure S34. HRMS, compound **9**; Table S1. Lipophilicity parameters of standard compounds; determined experimentally (R_M0_; mobile phase acetone/buffer Tris, pH 7.4) and with the literature values (logP_lit_).
