# Supplementary material for: Phosphoramidate Derivatives of Betulin, New Molecules with Promising Biological Activity: Synthesis and Characterization
Source: Molecules. 2026 Mar 11;31(6):935. doi: 10.3390/molecules31060935 (PMC13028633; doi:10.3390/molecules31060935)
Supplement: Supplementary file 1 [file molecules-31-00935-s001.zip › molecules-4160385-supplementary.pdf]

Supplementary Materials

Phosphoramidate derivatives of betulin, new molecules with promising biological activity: synthesis and characterization

Elwira Chrobak [1,*], Marta Świtalska [2], Marcel Madej [3,4], Joanna Wietrzyk [2], and Ewa Bębenek [1,*]

[1] Department of Organic Chemistry, Faculty of Pharmaceutical Sciences in Sosnowiec, Medical University of Silesia in Katowice, 4 Jagiellońska Str., 41-200 Sosnowiec, Poland; echrobak@sum.edu.pl (EC), ebebenek@sum.edu.pl (EB)
[2] Hirszfeld Institute of Immunology and Experimental Therapy, Polish Academy of Sciences, 12 Rudolfa Weigla Str., 53-114 Wrocław, marta.switalska@hirszfeld.pl (MŚ), joanna.wietrzyk@hirszfeld.pl (JW)
[3] Department of Molecular Biology, Faculty of Pharmaceutical Sciences in Sosnowiec, Medical University of Silesia, 40-055 Katowice, Poland.
[4] Silesia LabMed, Centre for Research and Implementation, Medical University of Silesia in Katowice, 18 Medykow Str., 40-752 Katowice, Poland mmarcel281297@gmail.com (MM)

Content:

Spectroscopic data for the compounds **3-5**, **6A**, **6B**, **7A**, **7B**, **8** and **9**.

Figure S1. ^{1}H NMR, compound **3**
Figure S2. ^{13}C NMR, compound **3**
Figure S3. ^{1}H NMR, compound **4**
Figure S4. ^{13}C NMR, compound **4**
Figure S5. ^{31}P NMR, compound **4**
Figure S6. HRMS, compound **4**
Figure S7. ^{1}H NMR, compound **5**
Figure S8. ^{13}C NMR, compound **5**
Figure S9. ^{31}P NMR, compound **5**
Figure S10. HRMS, compound **5**
Figure S11. ^{1}H NMR, compound **6A**
Figure S12. ^{13}C NMR, compound **6A**
Figure S13. ^{31}P NMR, compound **6A**
Figure S14. MS, compound **6A**
Figure S15. ^{1}H NMR, compound **6B**
Figure S16. ^{13}C NMR, compound **6B**
Figure S17. ^{31}P NMR, compound **6B**
Figure S18. HRMS, compound **6B**
Figure S19. ^{1}H NMR, compound **7A**
Figure S20. ^{13}C NMR, compound **7A**
Figure S21. ^{31}P NMR, compound **7A**
Figure S22. HRMS, compound **7A**
Figure S23. ^{1}H NMR, compound **7B**
Figure S24. ^{13}C NMR, compound **7B**
Figure S25. ^{31}P NMR, compound **7B**
Figure S26. HRMS, compound **7B**

Figure S27. ^{1}H NMR, compound **8**
Figure S28. ^{13}C NMR, compound **8**
Figure S29. ^{31}P NMR, compound **8**
Figure S30. HRMS, compound **8**
Figure S31. ^{1}H NMR, compound **9**
Figure S32. ^{13}C NMR, compound **9**
Figure S33. ^{31}P NMR, compound **9**
Figure S34. HRMS, compound **9**
Table S1. Lipophilicity parameters of standard compounds; determined experimentally (R_{M0}; mobile phase acetone:buffer Tris, pH 7.4) and literature values ($logP_{lit}$)

Spectroscopic data for the compounds **3-5**, **6A**, **6B**, **7A**, **7B**, **8** and **9**.

Compound 3: 3,28-di-*O*-acetyl-30-azidobetulin

Yield 91%; mp 189-190°C; R_f 0.27 (hexane:ethyl acetate, 8:1, *v/v*). ^{1}H NMR ($CDCl_3$, 600 MHz) δ (ppm): 0.79-0.81 (m, 1H, H-5), 0.85 (s, 3H, CH_3), 0.86 (s, 3H, CH_3), 0.99 (s, 3H, CH_3), 1.00 (s, 3H, CH_3), 1.06 (s, 3H, CH_3), 0.86-2.09 (m, 24H, CH, CH_2), 2.06 (s, 3H, OC(O)CH_3), 2.09 (s, 3H, CH_2OC(O)*CH_3*), 2.38 (m, 1H, H19), 3.78 (d, 2H, *J* = 11.4 Hz CH_2-30), 3.84 (d, 1H, ^{2}J = 11.4 Hz, H-28), 4.26 (d, 1H, ^{2}J = 11.4 Hz, H-28), 4.48 (m, 1H, H-3), 4.99 (s, 1H, H-29), 5.03 (s, 1H, H-29). ^{13}C NMR ($CDCl_3$, 150 MHz) δ (ppm): 14.7; 16.0; 16.2; 16.5; 18.2; 20.9; 21.1 (OC(O)**C**H_3); 21.4 (OC(O)**C**H_3) 23.7; 26.8; 27.0; 28.0; 29.7; 31.2; 34.1; 34.3; 37.1; 37.4; 37.8; 38.4; 40.9; 42.7; 44.1; 46.4; 49.8; 50.2; 55.3 (C-5); 62.5 (C-28); 80.9 (C-3); 111.7 (C-29); 148.5 (C-20); 171.1 (O**C**(O)CH_3); 171.6 (O**C**(O)CH_3).

Compound 4: 3,28-di-O-acetyl-30-(3-((dimethoxyphosphoryl)amino)prop-1-en-2-yl)betulin

Yield 91%; mp 110-112°C; R_f 0.07 (dichloromethane:ethanol, 40:1, *v/v*). ^{1}H NMR ($CDCl_3$, 600 MHz) δ (ppm): 0.79-0.81 (m, 1H, H-5), 0.85 (s, 3H, CH_3), 0.86 (s, 3H, CH), 0.86 (s, 3H, CH_3), 0.98 (s, 3H, CH_3), 1.04 (s, 3H, CH_3), 0.95-2.13 (m, 24H, CH, CH_2), 2.06 (s, 3H, OC(O)CH_3), 2.09 (s, 3H, CH_2OC(O)CH_3), 2.34 (m, 1H, H19), 3.48 (d, 2H, *J* = 11.4 Hz CH_2-30), 3.74 (s, 3H, OCH_3), 3.76 (s, 3H, OCH_3), 3.84 (d, 1H, ^{2}J = 11.4 Hz, H-28), 4.23 (d, 1H, ^{2}J = 11.4 Hz, H-28), 4.48 (m, 1H, H-3), 4.92 (s, 1H, H-29), 4.97 (s, 1H, H-29). ^{13}C NMR ($CDCl_3$, 150 MHz) δ (ppm): 14.7; 16.0; 16.2; 16.5; 18.2; 20.9; 21.1 (OC(O)**C**H_3); 21.4 (OC(O)**C**H_3) 23.7; 26.6; 27.0; 27.9; 29.7; 31.0; 31.3; 34.1; 34.3; 37.0; 37.45; 37.8; 38.4; 40.9; 42.6; 44.3; 46.3; 49.5; 50.2; 53.3 (OCH_3); 53.4 (OCH_3); 55.3; 62.4 (C-28); 80.9 (C-3); 107.7 (C-29); 152.4 (C-20); 171.1 (O**C**(O)CH_3); 171.6 (O**C**(O)CH_3). ^{31}P NMR ($CDCl_3$, 243 MHz) δ (ppm): 11.17. HRMS (APCI) m/z (neg): 648.4019; $C_{36}H_{59}NO_7P$ [M-H] (Calculated 648.4029).

Compound 5: 30-(3-((dimethoxyphosphoryl)amino)prop-1-en-2-yl)betulin

Yield 82%; mp 150-152°C; R_f 0.29 (dichloromethane:ethanol, 15:1, *v/v*). ^{1}H NMR ($CDCl_3$, 600 MHz) δ (ppm): 0.67-0.69 (m, 1H, H-5), 0.75 (s, 3H, CH_3), 0.81 (s, 3H, CH_3), 0.97 (s, 3H, CH_3), 0.98 (s, 3H, CH_3), 1.01 (s, 3H, CH_3), 0.86-2.15 (m, 26H, CH, CH_2), 2.28 (m, 1H, H19), 3.18 (m, 1H, H-3), 3.30 (d, *J*=10.8Hz, 1H, H-28), 3.46 (m, 2H, CH_2-30), 3.71 (s, 3H, OCH_3), 3.72 (s, 3H, OCH_3), 3.78 (d, *J*=10.8Hz, 1H, H-28), 4.90 (s, 1H, H-29), 4.93 (s, 1H, H-29). ^{13}C NMR ($CDCl_3$, 150 MHz)

δ (ppm): 14.8; 15.4; 16.0; 16.1; 18.3; 20.9; 26.7; 27.0; 27.3; 28.0; 29.2; 31.5; 33.8; 34.2; 37.1; 37.2; 38.7; 38.9; 40.89; 40.9; 42.7; 46.7; 47.8; 49.4; 50.3; 53.2 (d, OCH_3); 53.3 (d, OCH_3); 55.3 (C-5); 60.3 (C-28); 79.0 (C-3); 107.5 (C-29); 152.6 (C-20). ^{31}P NMR ($CDCl_3$, 243 MHz) δ (ppm): 11.23. HRMS (APCI) m/z (neg): 564.3815; $C_{32}H_{55}NO_5P$ [M-H] (Calculated 564.3818).

Compound 6A: **30-(3-((dimethoxyphosphoryl)amino)prop-1-en-2-yl)-3,28-di-*O*-propynoyl betulin**

Yield 26%; mp 92-94°C; R_f 0.44 (dichloromethane:ethanol, 15:1, *v*/*v*). ^{1}H NMR ($CDCl_3$, 600 MHz) δ (ppm): 0.70-0.72 (m, 1H, H-5), 0.79 (s, 3H, CH_3), 0.81 (s, 3H, CH_3), 0.82 (s, 3H, CH_3), 0.91(s, 3H, CH_3), 0.96 (s, 3H, CH_3), 0.76-2.30- (m, 24H, CH, CH_2), 2.57 (m, 1H, H19), 2.73 (s, 1H, ≡C-H), 2.84 (s, 1H, ≡C-H), 3.40 (m, 2H, CH_2-30), 3.65 (s, 3H, OCH_3), 3.67 (s, 3H, OCH_3), 3.89 (d, *J*=11.4Hz, 1H, H-28), 4.29 (d, *J*=11.4Hz, 1H, H-28), 4.53 (m, 1H, H-3), 4.84 (s, 1H, H-29), 4.89 (s, 1H, H-29). ^{13}C NMR ($CDCl_3$, 150 MHz) δ (ppm): 14.7; 16.0; 16.2; 16.5; 18.1; 20.9; 23.5; 24.1; 24.5; 24.8; 25.2; 26.6; 26.9; 27.9; 29.6; 29.7; 31.2; 32.2; 32.8; 34.1; 34.2; 37.0; 37.5; 37.9; 38.4; 40.9; 42.7; 44.4; 46.4; 49.5; 50.1; 53.2 (d, OCH_3); 53.3 (d, OCH_3); 55.3 (C-5); 64.5 (C-28); 74.7; 74.9; 83.6 (C-3); 107.8 (C-29); 153.8 (C-20); 153.2 (2xC(O)O). ^{31}P NMR ($CDCl_3$, 243 MHz) δ (ppm): 11.10. TOF MS (ES+) m/z: 670.3843; $C_{38}H_{55}NO_7P$ [M+H] (Calculated 670.3853.

Compound 6B: 30-(3-((dimethoxyphosphoryl)amino)prop-1-en-2-yl)-28-*O*-propynoyl betulin

Yield 56%; mp 119-122°C; R_f 0.28 (dichloromethane:ethanol, 15:1, *v*/*v*). ^{1}H NMR ($CDCl_3$, 600 MHz) δ (ppm): 0.68-0.69 (m, 1H, H-5), 0.77 (s, 3H, CH_3), 0.83 (s, 3H, CH_3), 0.98 (s, 3H, CH_3), 0.99 (s, 3H, CH_3), 1.03 (s, 3H, CH_3), 0.80-2.16 (m, 24H, CH, CH_2), 2.33 (m, 1H, H19), 2.93 (s, 1H, ≡C-H), 3.20 (m, 1H, H-3), 3.48(m, 2H, CH_2-30), 3.73 (s, 3H, OCH_3), 3.74 (s, 3H, OCH_3), 3.97 (d, *J*=11.4Hz, 1H, H-28), 4.36 (d, *J*=11.4Hz, 1H, H-28), 4.91 (s, 1H, H-29), 4.97 (s, 1H, H-29). ^{13}C NMR ($CDCl_3$, 150 MHz) δ (ppm): 14.8; 15.4; 16.0; 16.1; 18.3; 20.9; 26.6; 26.9; 27.3; 28.0; 29.6; 34.2; 37.1; 37.5; 37.6; 38.7; 38.9; 40.9; 42.7; 44.5; 46.3; 49.5; 50.3; 53.3 (d, OCH_3); 53.3 (d, OCH_3); 55.3 (C-5); 64.5 (C-28); 74.9; 79.0 (C-3); 107.8 (C-29); 153.2 (C-20); 161.5 (C(O)O). ^{31}P NMR ($CDCl_3$, 243 MHz) δ (ppm): 11.19. HRMS (ESI) m/z (neg): 616.3770; $C_{32}H_{55}NO_5P$ [M-H] (Calculated 616.3767).

Compound 7A: 30-(3-((dimethoxyphosphoryl)amino)prop-1-en-2-yl)-3,28-di-*O*-but-2-ynoyl betulin

Yield 16%; mp 99-102°C; R_f 0.63 (dichloromethane:ethanol, 15:1, *v*/*v*). ^{1}H NMR ($CDCl_3$, 600 MHz) δ (ppm): 0.70-0.71 (m, 1H, H-5), 0.77 (s, 3H, CH_3), 0.80 (s, 3H, CH_3), 0.81 (s, 3H, CH_3), 0.90 (s, 3H, CH_3), 0.95 (s, 3H, CH_3), 0.86-2.30 (m, 23H, CH, CH_2), 2.59 (m, 1H, H19), 1.92 (s, 3H, ≡C-CH_3), 1.93 (s, 3H, ≡C-CH_3), 3.35-3.46 (m, 2H, CH_2-30), 3.65 (s, 3H, OCH_3), 3.67 (s, 3H, OCH_3), 3.85 (d, *J*=10.8Hz, 1H, H-28), 4.24 (d, *J*=10.8Hz, 1H, H-28), 4.61 (m, 1H, N-H), 4.49-4.51 (m, 1H, H-3), 4.83 (s, 1H, H-29), 4.88 (s, 1H, H-29). ^{13}C NMR ($CDCl_3$, 150 MHz) δ (ppm): 3.88 (2xCH_3); 14.0; 14.7; 16.0; 16.1; 16.5; 18.1; 20.9; 27.0; 27.9; 29.6; 29.7; 34.1; 34.3; 37.0; 37.5; 37.9; 38.4; 40.9; 42.6; 44.4; 46.4; 49.5; 50.2; 53.2 (d, OCH_3); 53.3 (d, OCH_3); 55.4 (C-5); 63.8 (C-28); 72.4; 72.8; 82.7; 85.0; 85.7; 107.8 (C-29); 153,9; 154.3 (C-20); 165.4 (C(O)O). ^{31}P NMR ($CDCl_3$, 243 MHz) δ (ppm): 11.15. HRMS (ESI) m/z (neg): 696.4029; $C_{40}H_{59}NO_7P$ [M-H] (Calculated 696.4016).

Compound 7B: 30-(3-((dimethoxyphosphoryl)amino)prop-1-en-2-yl)-28-*O*-but-2-ynoyl betulin

Yield 35%; mp 109-110°C; R_f 0.34 (dichloromethane:ethanol, 15:1, *v/v*). ^{1}H NMR ($CDCl_3$, 600 MHz) δ (ppm): 0.68-0.70 (m, 1H, H-5), 0.78 (s, 3H, CH_3), 0.83 (s, 3H, CH_3), 0.98 (s, 3H, CH_3), 0.99 (s, 3H, CH_3), 1.03 (s, 3H, CH_3), 0.86-2.11 (m, 24H, CH, CH_2), 2.01 (s, 3H, ≡C-CH_3), 2.34 (m, 1H, H19), 3.20 (m, 1H, H-3), 3.48 (m, 2H, CH_2-30), 3.74 (s, 3H, OCH_3), 3.75 (s, 3H, OCH_3), 3.94 (d, *J*=11.4Hz, 1H, H-28), 4.32 (d, *J*=11.4Hz, 1H, H-28), 4.92 (s, 1H, H-29), 4.97 (s, 1H, H-29). ^{13}C NMR ($CDCl_3$, 150 MHz) δ (ppm): 3.9; 14.8; 15.4; 16.0; 16.1; 18.3; 20.9; 26.6; 27.0; 27.3; 28.0; 29.6; 34.1; 34.2; 37.0; 37.5; 38.7; 38.9; 40.9; 42.7; 44.2; 46.4; 49.5; 50.3; 53.3 (d, OCH_3); 53.4 (d, OCH_3); 55.3 (C-5); 63.8(C-28); 72.5; 79.0 (C-3); 85.8; 107.6 (C-29); 152.2 (C-20); 154.3 (C(O)O). ^{31}P NMR ($CDCl_3$, 243 MHz) δ (ppm): 11.19. HRMS (ESI) m/z (neg): 630.3933; $C_{36}H_{57}NO_6P$ [M-H] (Calculated 630.3924).

Compound 8: 30-(3-((dimethoxyphosphoryl)amino)prop-1-en-2-yl)betulonic acid

Yield 32%; mp 223-225°C; R_f 0.20 (dichloromethane:ethanol, 30:1, *v/v*). ^{1}H NMR ($CDCl_3$, 600 MHz) δ (ppm): 0.94 (s, 3H, CH_3), 0.97 (s, 3H, CH_3), 1.01 (s, 3H, CH_3), 1.03 (s, 3H, CH_3), 1.09 (s, 3H, CH_3), 0.90-2.55 (m, 22H, CH, CH_2), 2.90 (m, 1H, H19), 3.50 (m, 2H, CH_2-30), 3.73 (s, 3H, OCH_3), 3,74 (s, 3H, OCH_3), 4.93 (s, 1H, H-29), 4.96 (s, 1H, H-29). ^{13}C NMR ($CDCl_3$, 150 MHz) δ (ppm): 14.6; 15.8; 16.0; 18.4; 19.6; 21.0; 21.5; 26.7; 27.0; 29.7; 31.0; 32.0; 33.6; 34.1; 36.7; 36.9; 38.3; 39.6; 40.6; 42.4; 45.8; 47.3; 49.8; 50.4; 53.2 (d, OCH_3); 53.3 (d, OCH_3); 54.9 (C-5); 56.15; 107.4 (C-29); 153.2 (C-20); 179.8 (COOH); 218.2 (C-3). ^{31}P NMR ($CDCl_3$, 243 MHz) δ (ppm): 11.69. HRMS (APCI) m/z (neg): 576.3440; $C_{32}H_{51}NO_6P$ [M-H] (Calculated 576.3454).

Compound 9: 30-(3-((dimethoxyphosphoryl)amino)prop-1-en-2-yl)betulinic acid

Yield 76%; mp 260-262°C; R_f 0.16 (chloroform:ethanol; 15:1, *v/v*). ^{1}H NMR ($CDCl_3$, 600 MHz) δ (ppm): 0.67-0.69 (m, 1H, H-5), 0.75 (s, 3H, CH_3), 0.81 (s, 3H, CH_3), 0.91 (s, 3H, CH_3), 0.96 (s, 3H, CH_3), 0.97 (s, 3H, CH_3), 0.80-2.30 (m, 24H, CH, CH_2), 2.86 (m, 1H, H19), 3.19 (m, 1H, H-3), 3.45 (m, 2H, CH_2-30), 3.71 (m, 3H, OCH_3), 3.73 (m, 3H, OCH_3), 4.90 (s, 1H, H-29), 4.94 (s, 1H, H-29). ^{13}C NMR ($CDCl_3$, 150 MHz) δ (ppm): 14.7; 15.4; 16.0; 16.1; 16.2; 18.3; 21.0; 26.9; 27.3; 28.0; 29.7; 32.1; 32.5; 34.3; 36.8; 37.2; 38.2; 38.7; 38.9; 40.7; 42.4; 43.0; 45.7; 49.8; 50.5; 53.2 (d; OCH_3); 53.3 (d; OCH_3); 55.3 (C-5); 56.2; 79.0 (C-3); 107.4 (C-29); 153.2 (C-20); 180.0 (COOH);. ^{31}P NMR ($CDCl_3$, 243 MHz) δ (ppm): 11.71 HRMS (APCI) m/z (neg): 578.3618; $C_{32}H_{51}NO_6P$ [M-H] (Calculated 578.3610

Compound 3: 3,28-di-O-acetyl-30-azidobetulin

Figure S1. ^{1}H NMR

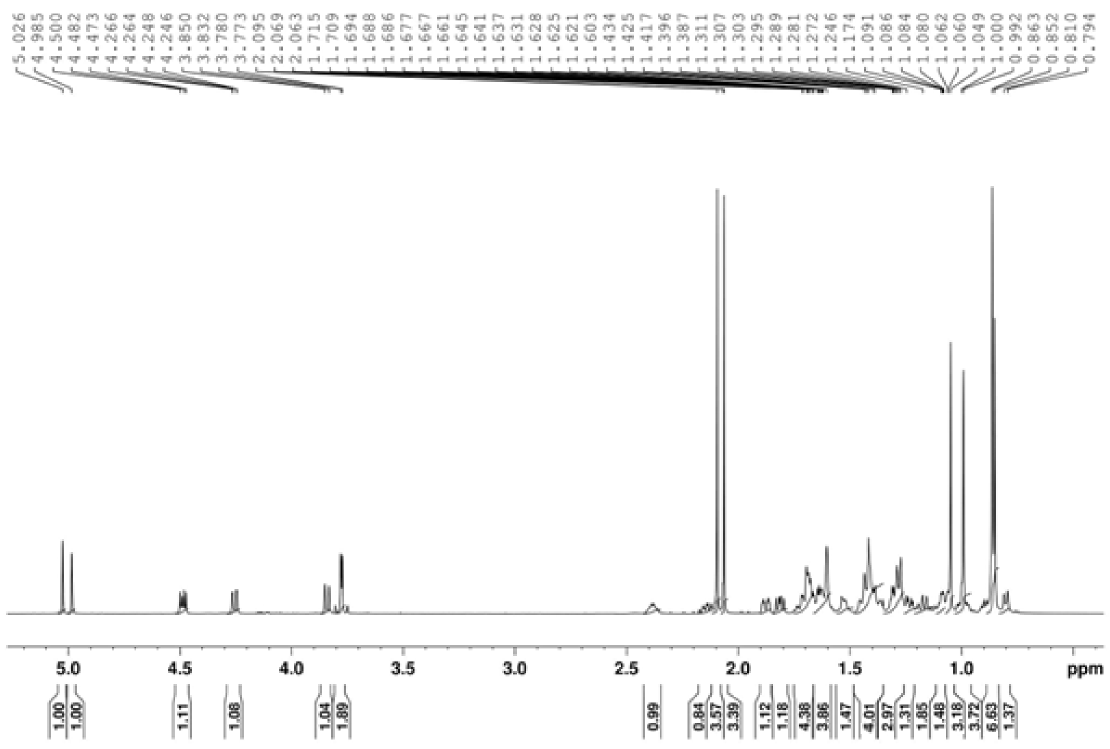

Figure S2. ^{13}C NMR, compound **3**

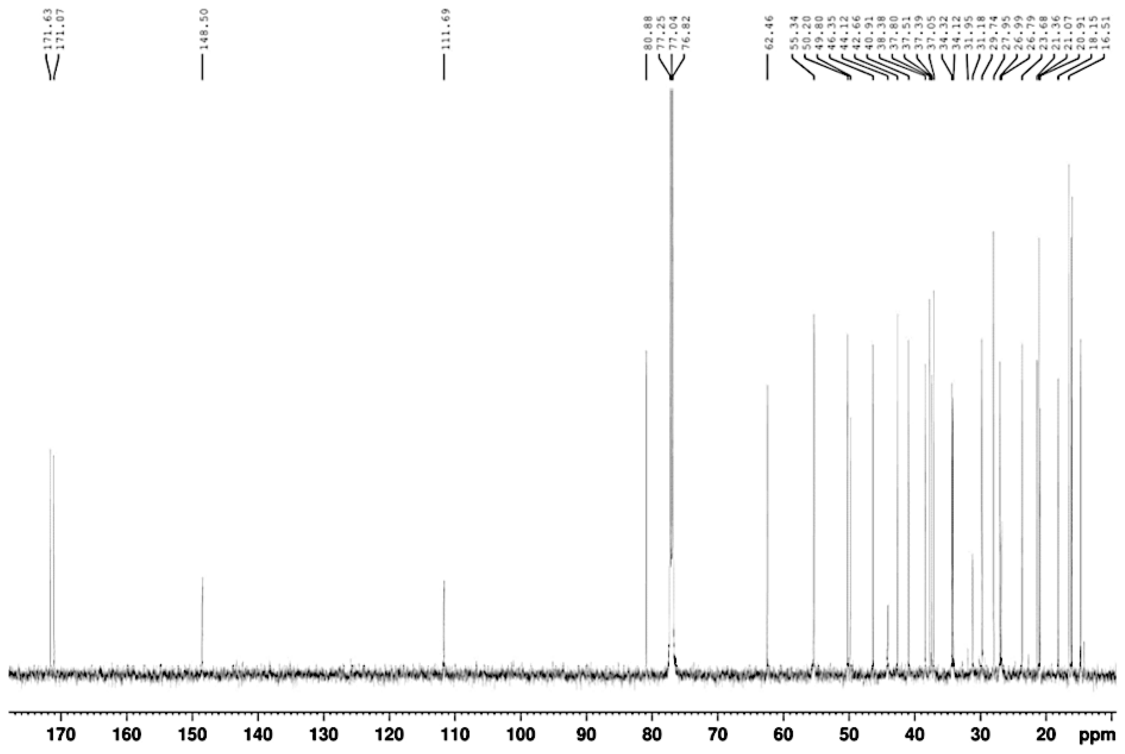

Compound 4: 3,28-di-O-acetyl- 30-(3-((dimethoxyphosphoryl)amino)prop-1-en-2-yl)betulin

Figure S3. ^{1}H NMR, compound **4**

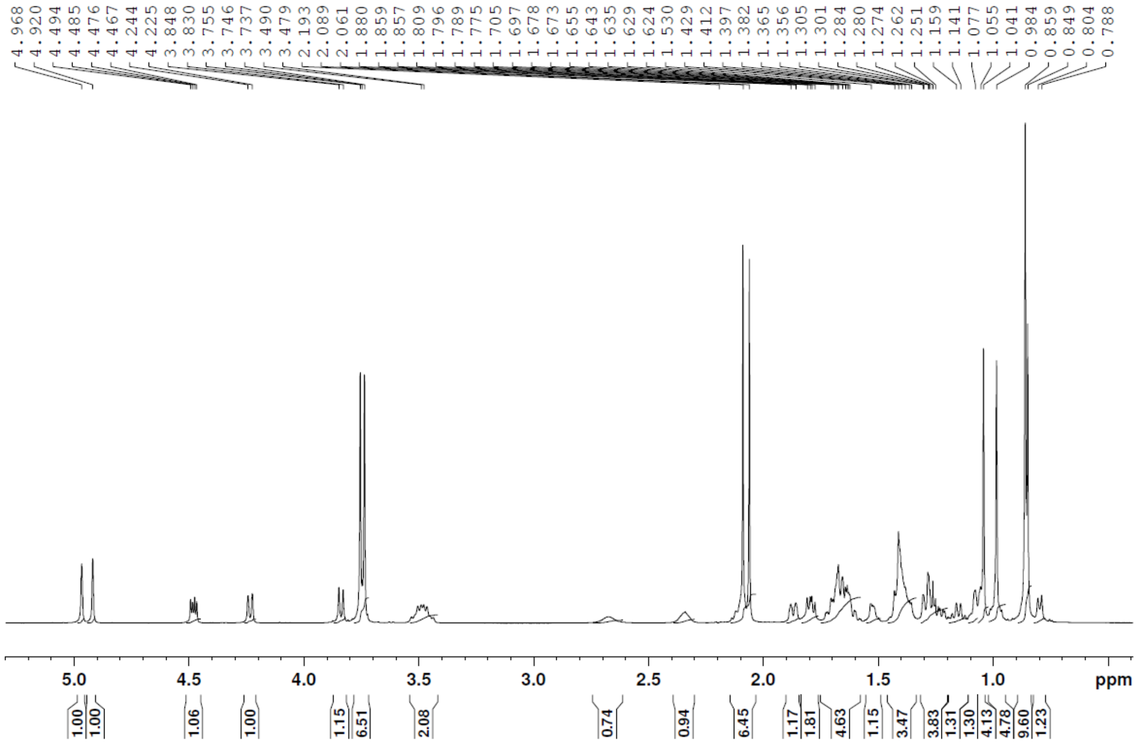

Figure S4. ^{13}C NMR, compound **4**

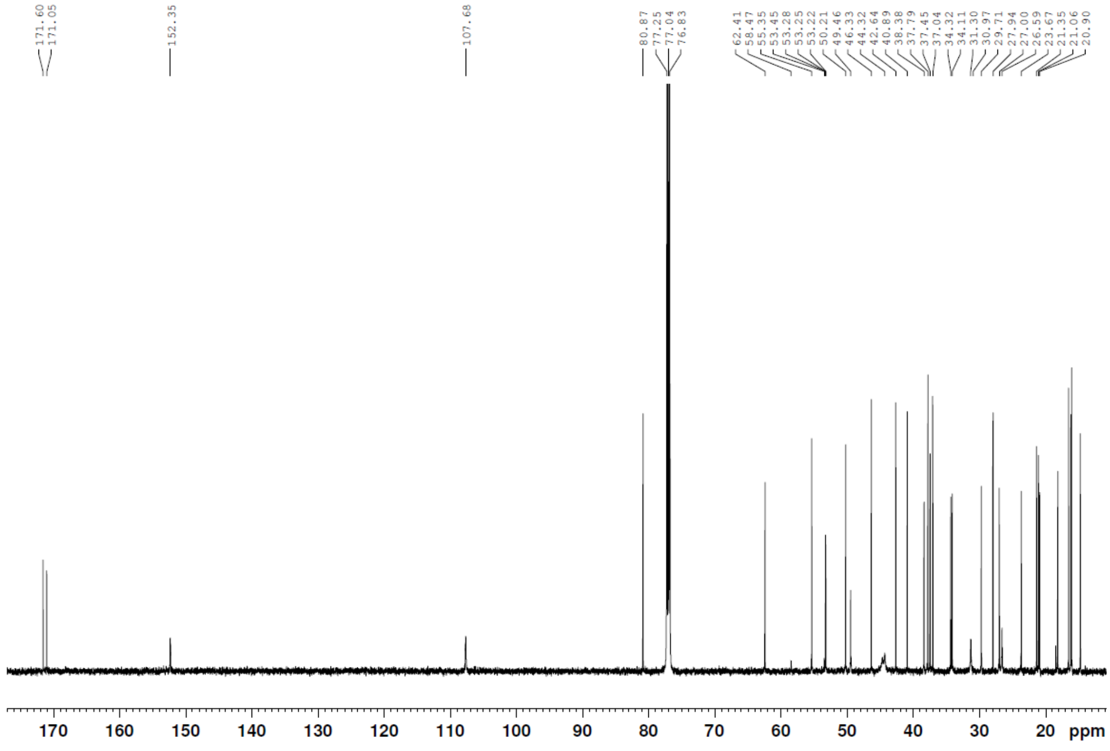

Figure S5. ^{31}P NMR, compound **4**

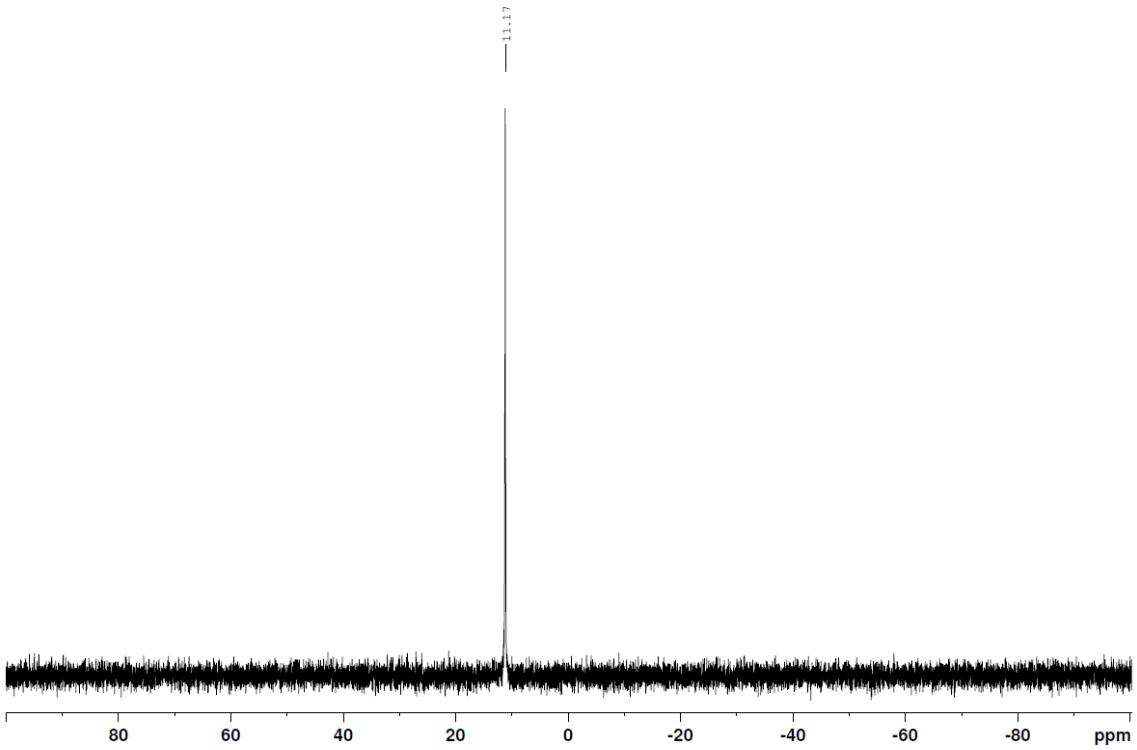

Figure S6. HRMS, compound **4**

Analysis Name	D:\Data\ECh176.d			
Method	low_mass.m	Operator	KM	
Sample Name	TM Low concentration	Instrument	impact II	1825265.10082
Comment				

Acquisition Parameter

Source Type	APCI	Ion Polarity	Negative	Set Nebulizer	2.0 Bar
Focus	Active	Set Capillary	4000 V	Set Dry Heater	200 °C
Scan Begin	100 m/z	Set End Plate Offset	-500 V	Set Dry Gas	5.0 l/min
Scan End	1000 m/z	Set Charging Voltage	2000 V	Set Divert Valve	Source
		Set Corona	2000 nA	Set APCI Heater	450 °C

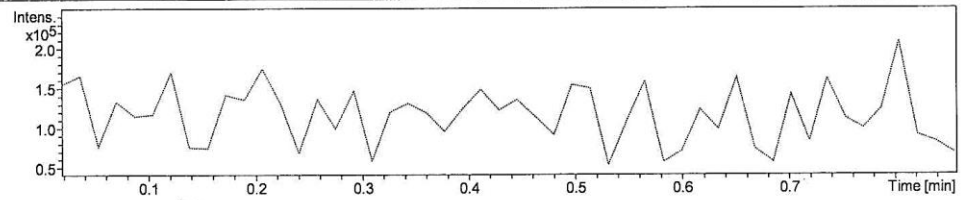

-MS, 0.0-0.9min #1-50

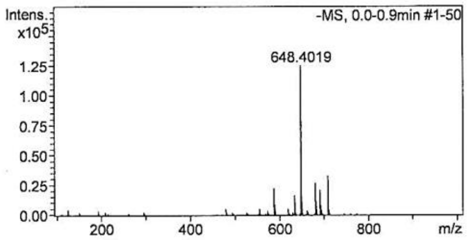

#	m/z	Res.	S/N	I	I %	FWHM
1	588.3813	37627	1672.2	22703	18.2	0.0156
2	634.3865	36843	1164.6	16762	13.4	0.0172
3	648.4019	47124	8436.4	124918	100.0	0.0138
4	681.3995	41784	1792.6	27607	22.1	0.0163
5	691.4077	38075	1361.3	21094	16.9	0.0182
6	708.4227	43681	2163.6	33357	26.7	0.0162

Compound 5: 30-(3-((dimethoxyphosphoryl)amino)prop-1-en-2-yl)betulin

Figure S7. ^{1}H NMR, compound **5**

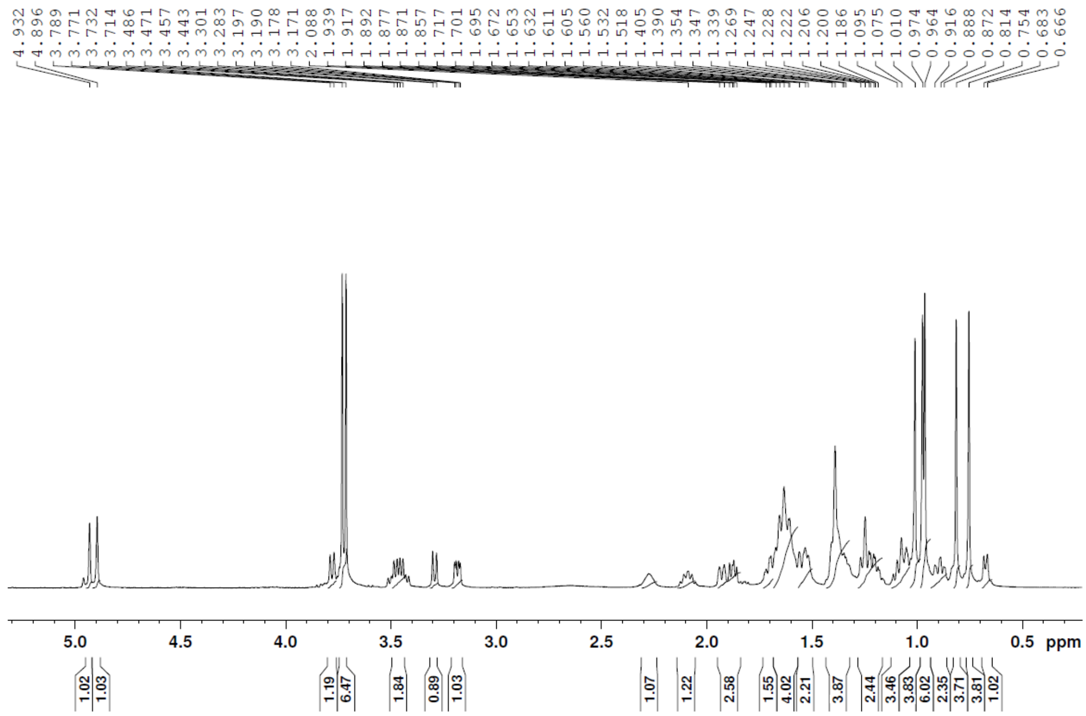

Figure S8. ^{13}C NMR, compound **5**

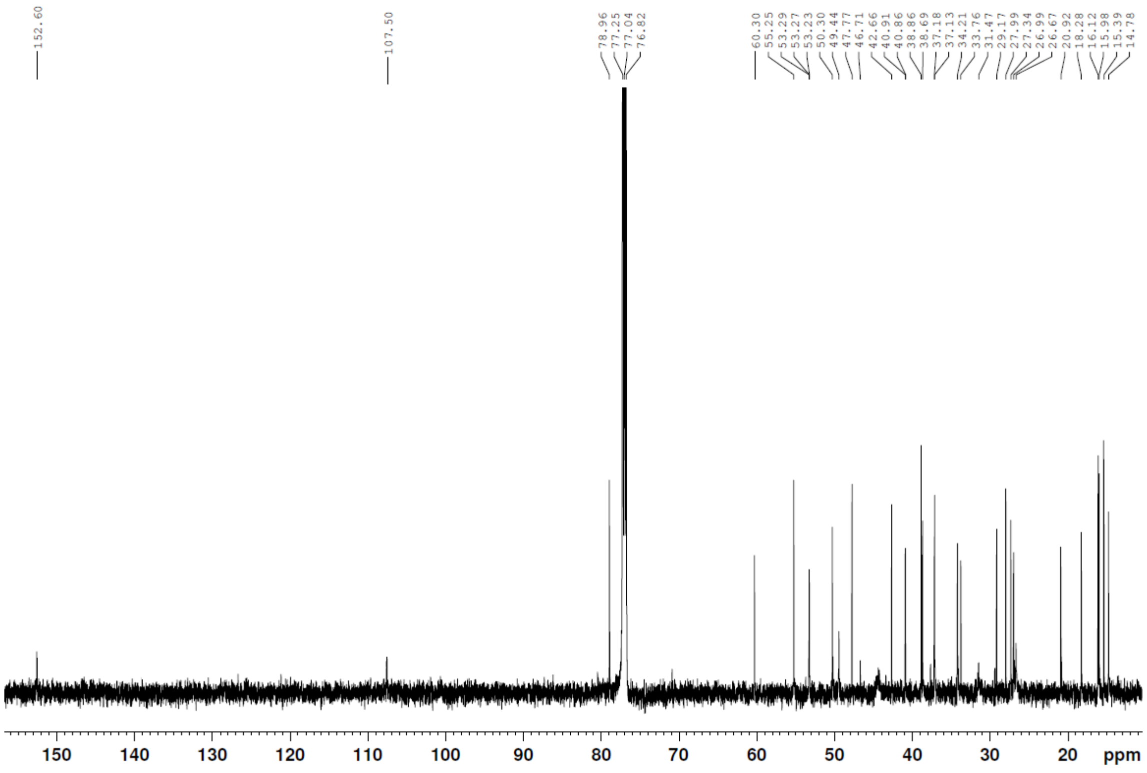

Figure S9. ^{31}P NMR, compound **5**

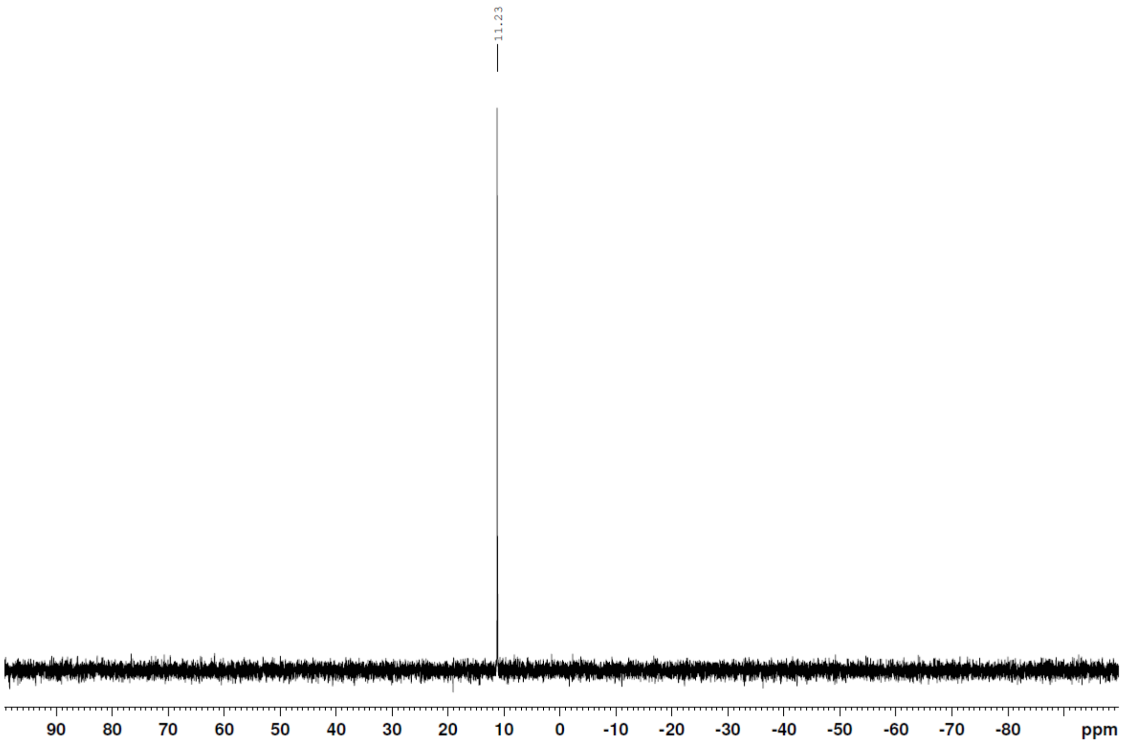

Figure S10. HRMS, compound **5**

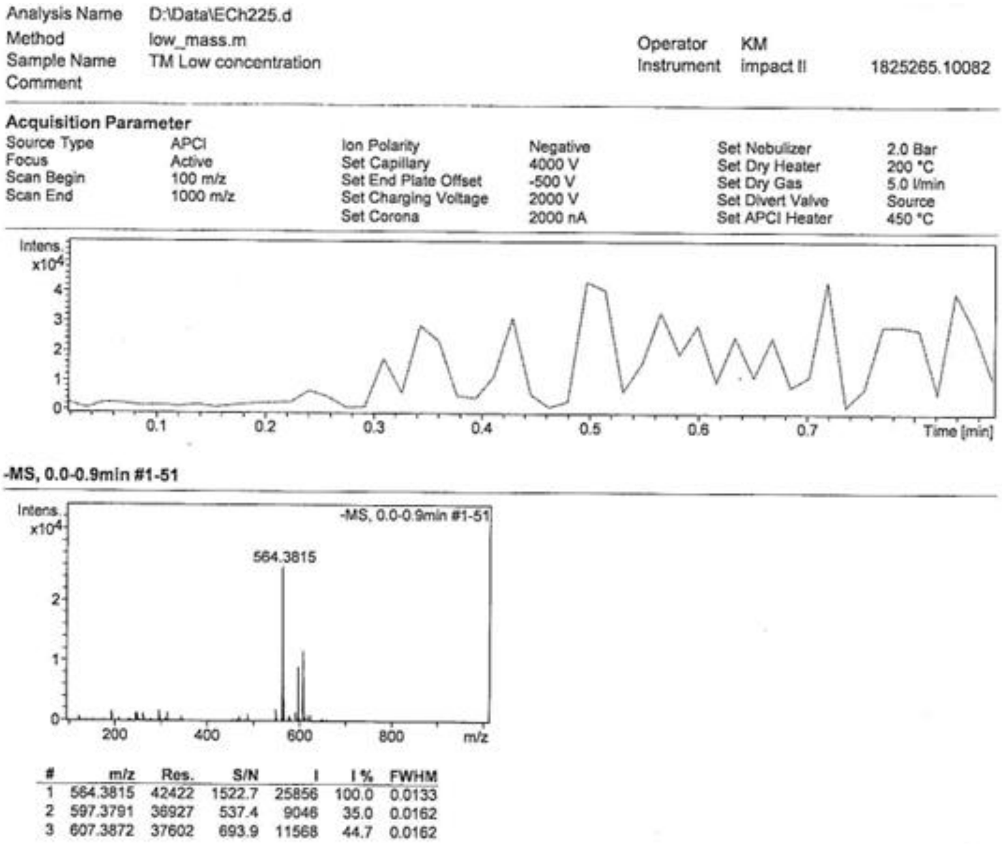

Compound 6A: **30-(3-((dimethoxyphosphoryl)amino)prop-1-en-2-yl)-3,28-di-*O*-propynoyl betulin**

Figure S11. ^{1}H NMR, compound **6A**

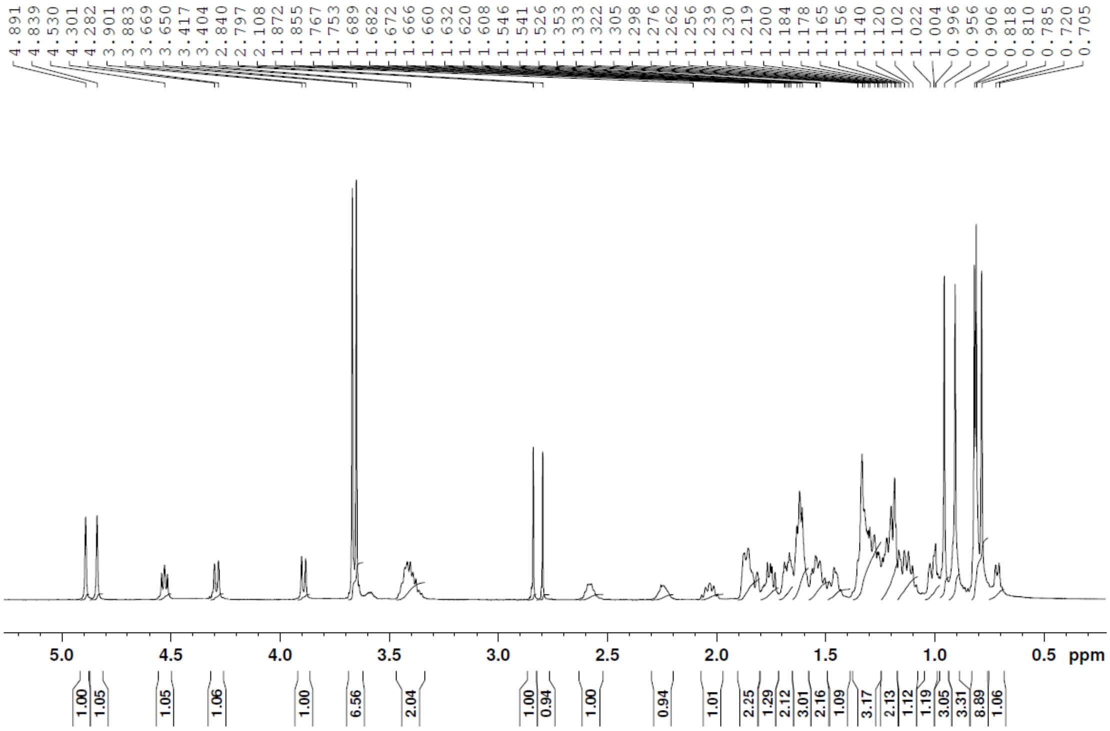

Figure S12. ^{13}C NMR, compound **6A**

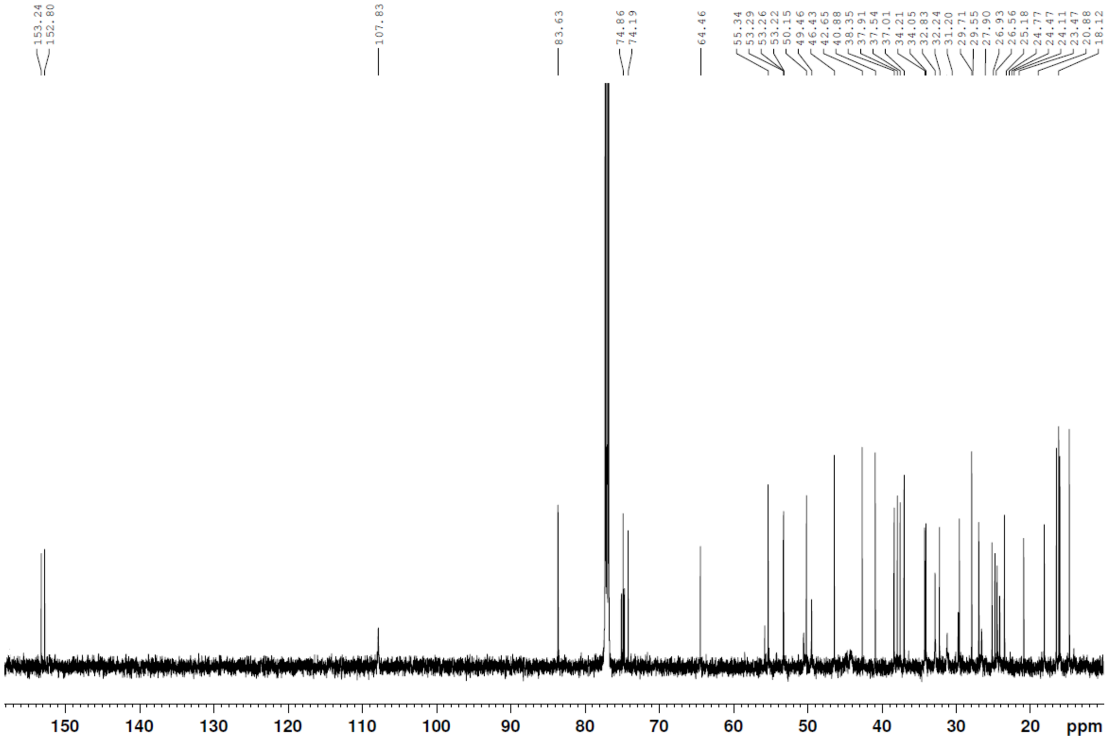

Figure S13. ^{31}P NMR, compound **6A**

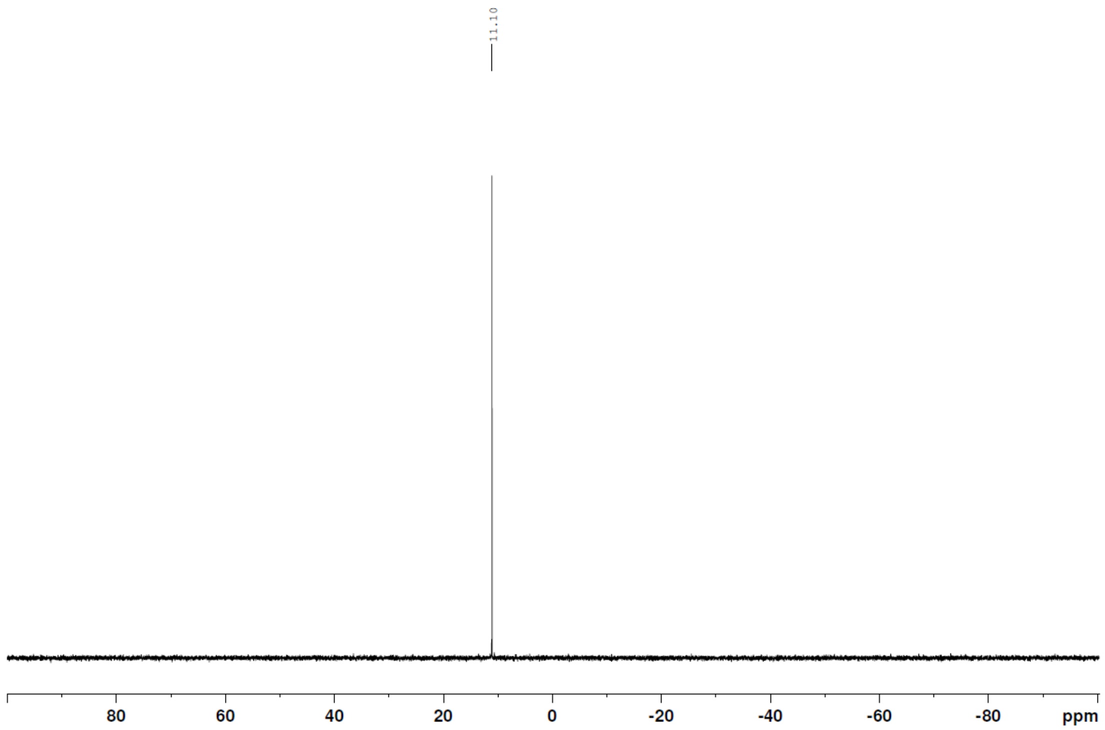

Figure S14. MS, compound **6A**

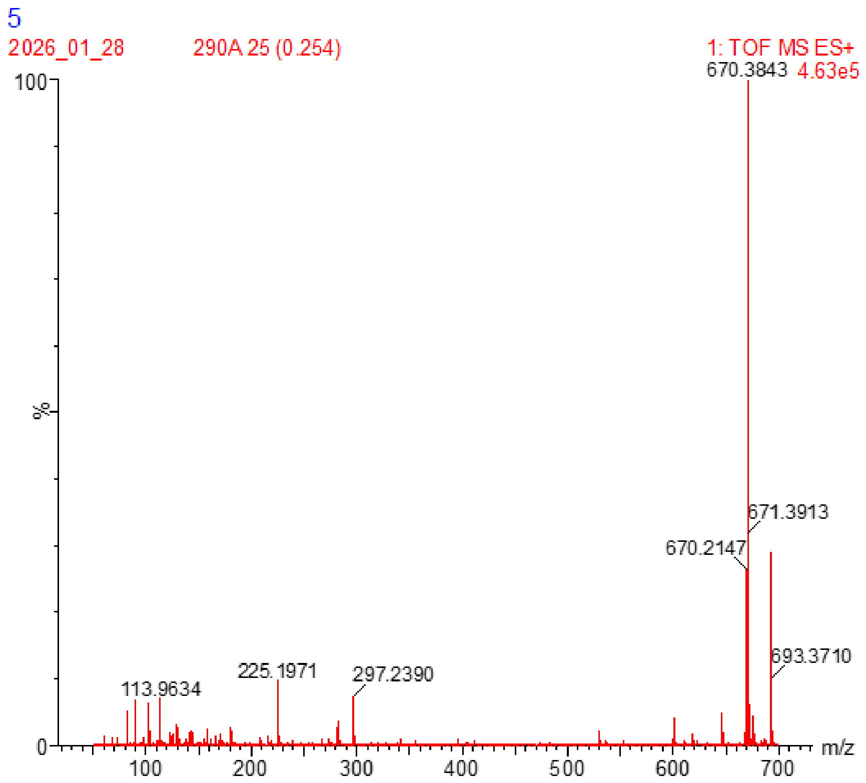

Compound 6B: 30-(3-((dimethoxyphosphoryl)amino)prop-1-en-2-yl)-28-*O*-propynoyl betulin

Figure S15. ^{1}H NMR, compound **6B**

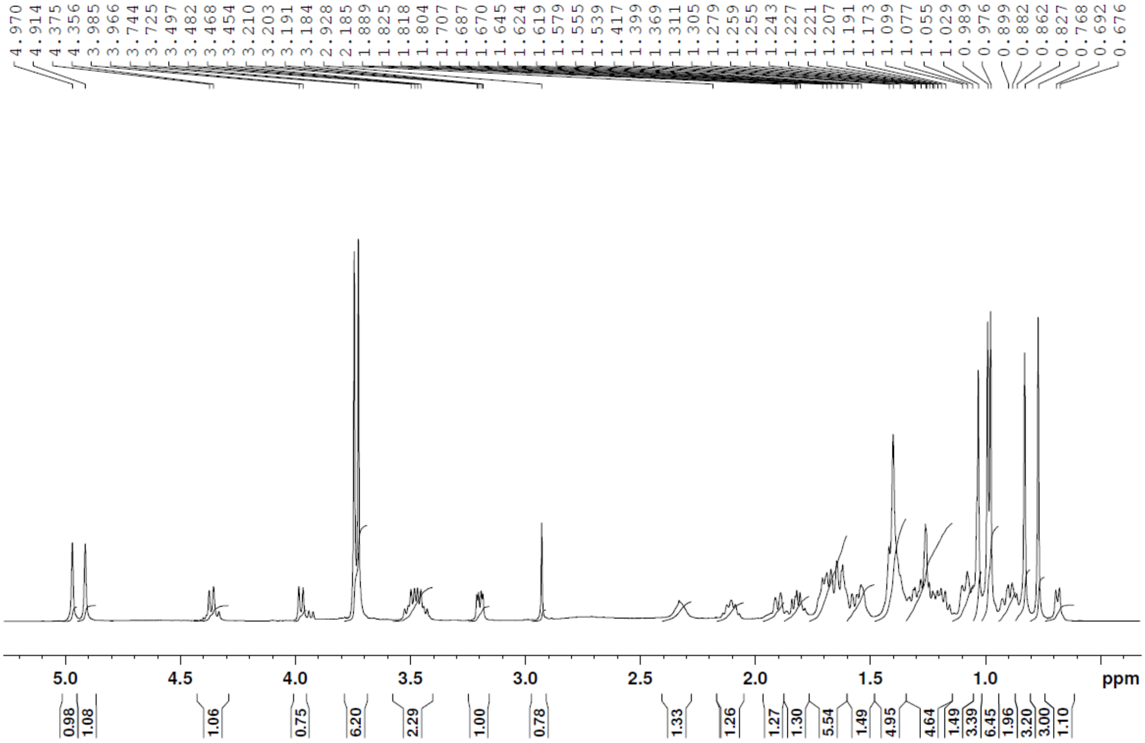

Figure S16. ^{13}C NMR, compound **6B**

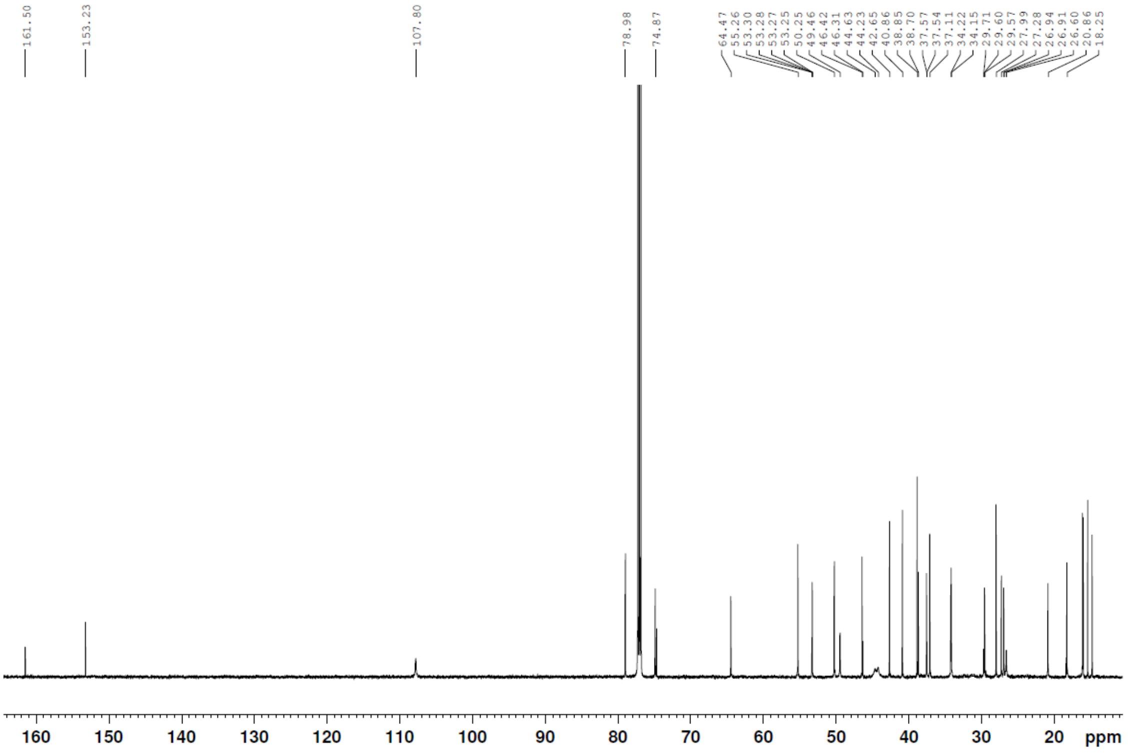

Figure S17. ^{31}P NMR, compound **6B**

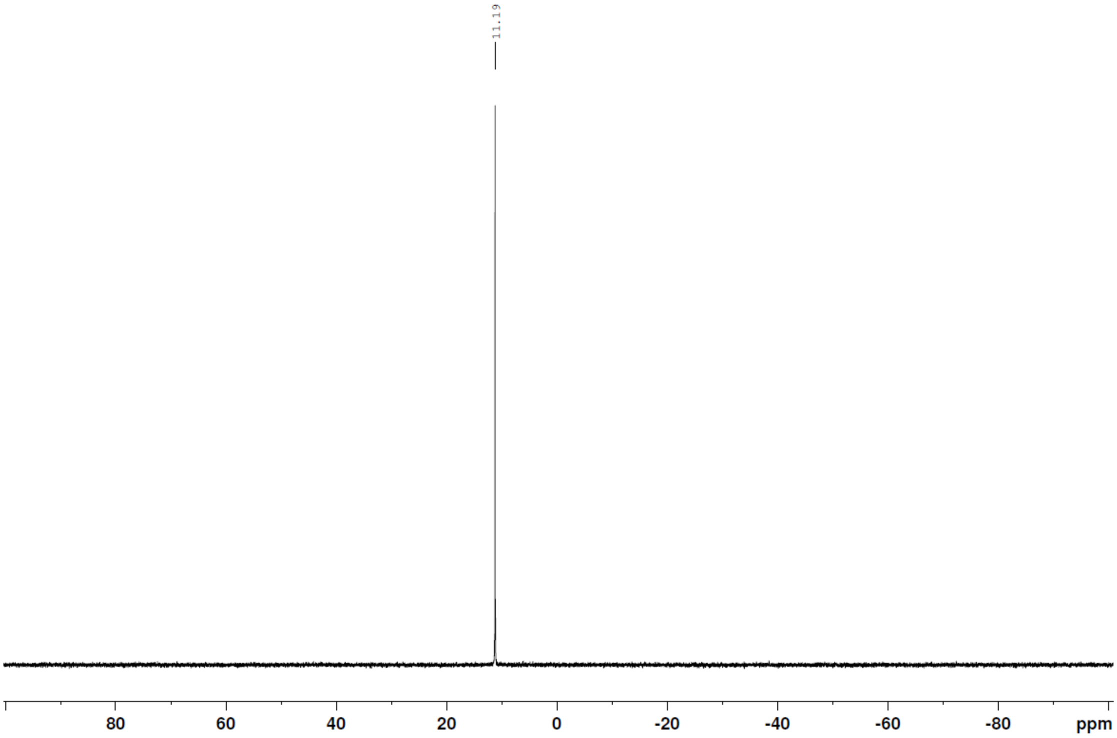

Figure S18. HRMS, compound **6B**

Analysis Name	D:\Data\specs\PM_20251103\performance 2\ECH 290B.d			
Method	low_mass_positive.m	Operator	KM	
Sample Name	Tune_neg_high_post	Instrument	impact II	1825265.10082
Comment				

Acquisition Parameter

Source Type	ESI	Ion Polarity	Negative	Set Nebulizer	0.3 Bar
Focus	Active	Set Capillary	2500 V	Set Dry Heater	200 °C
Scan Begin	100 m/z	Set End Plate Offset	-500 V	Set Dry Gas	3.0 l/min
Scan End	1000 m/z	Set Charging Voltage	2000 V	Set Divert Valve	Source
		Set Corona	0 nA	Set APCI Heater	0 °C

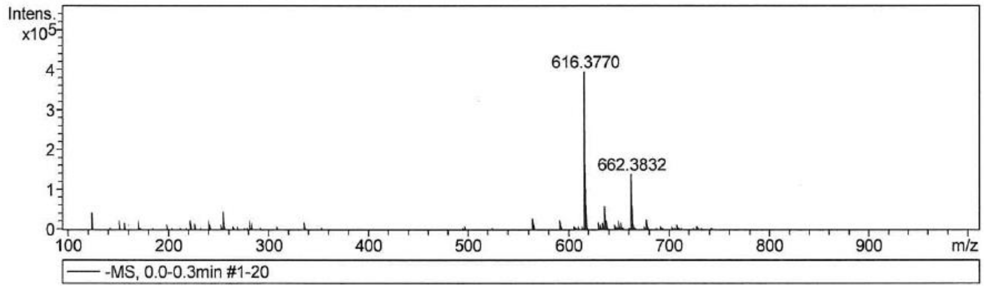

#	m/z	Res.	S/N	I	I %	FWHM
1	616.3770	22932	12096.1	393931	100.0	0.0269
2	662.3832	18869	4216.5	140018	35.5	0.0351

Compound 7A: 30-(3-((dimethoxyphosphoryl)amino)prop-1-en-2-yl)-3,28-di-*O*-but-2-ynoyl betulin

Figure S19. ^{1}H NMR, compound **7A**

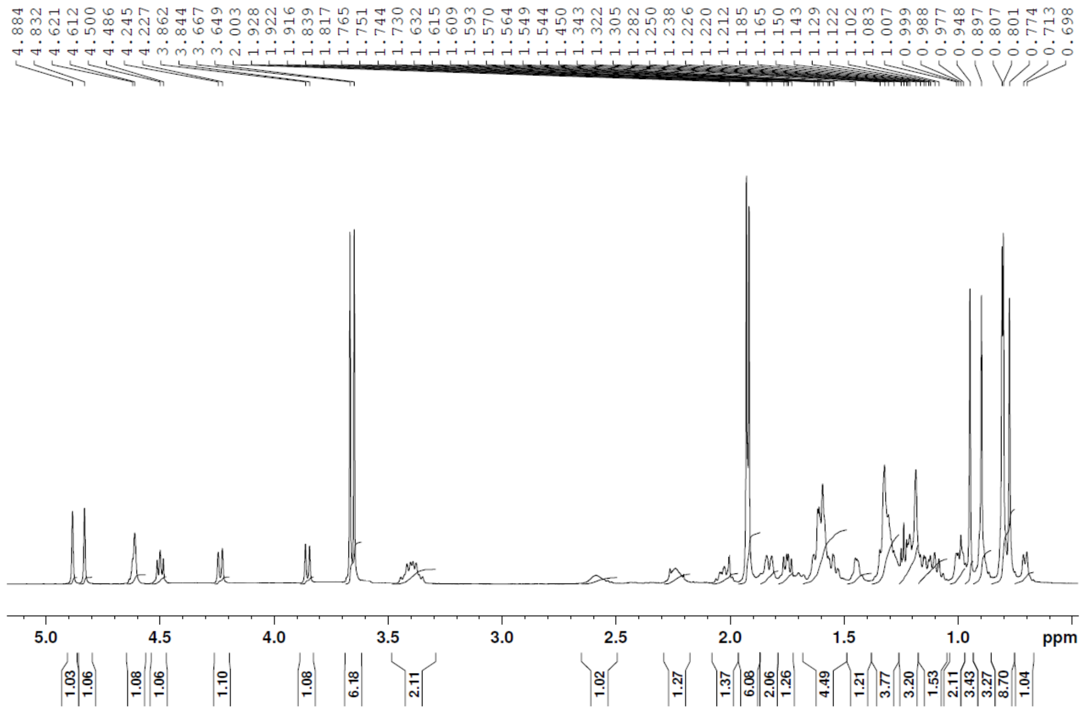

Figure S20. ^{13}C NMR, compound **7A**

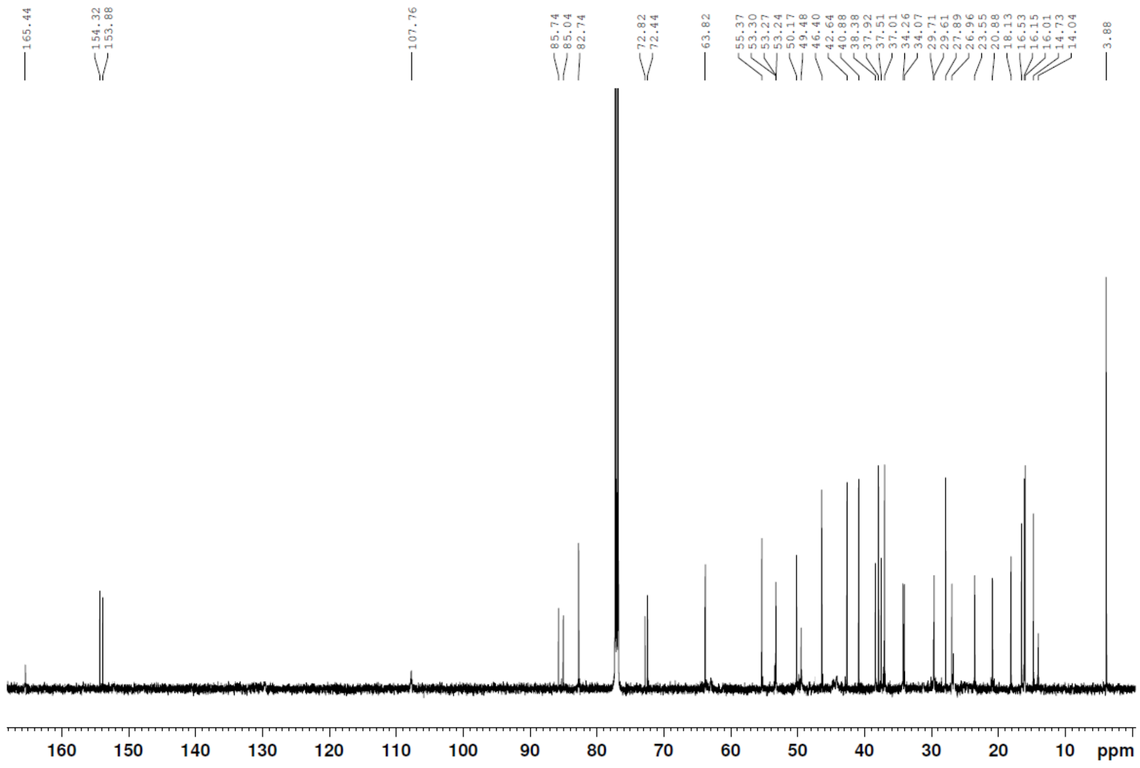

Figure S21. ^{31}P NMR, compound **7A**

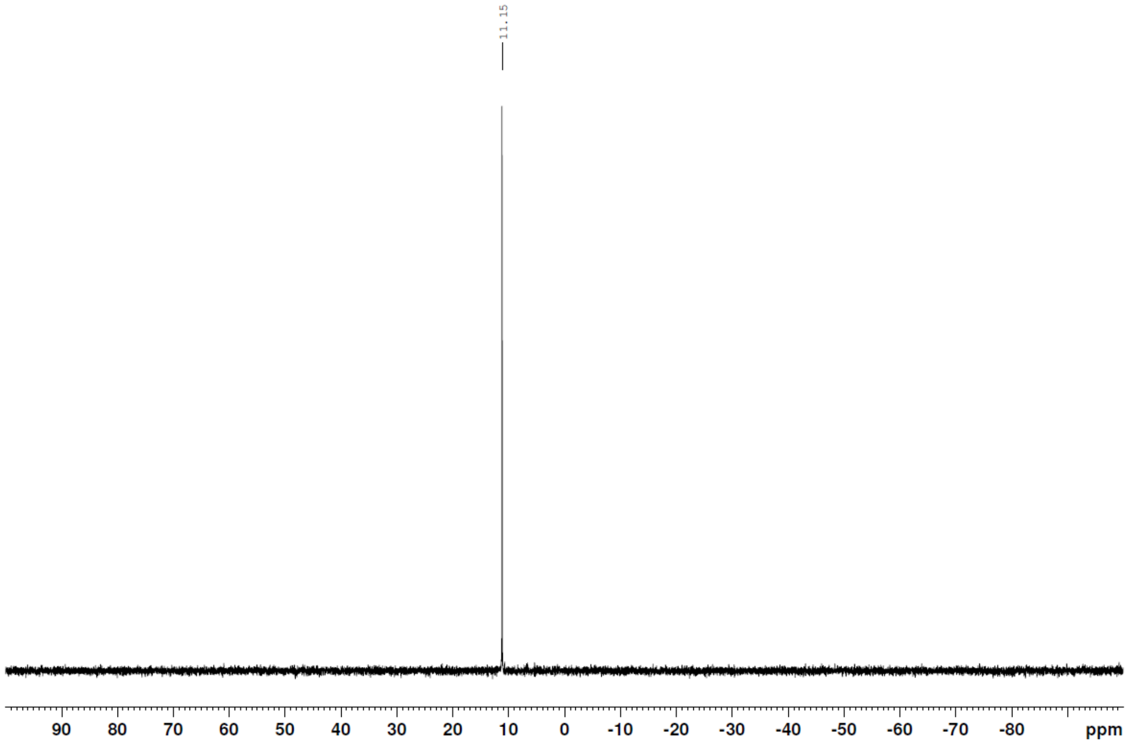

Figure S22. HRMS, compound **7A**

Analysis Name	D:\Data\specs\PM_20251103\performance 2\ECH 292A.d			
Method	low_mass_positive.m	Operator	KM	
Sample Name	Tune_neg_high_post	Instrument	impact II	1825265.10082
Comment				

Acquisition Parameter

Source Type	ESI	Ion Polarity	Negative	Set Nebulizer	0.3 Bar
Focus	Active	Set Capillary	2500 V	Set Dry Heater	200 °C
Scan Begin	100 m/z	Set End Plate Offset	-500 V	Set Dry Gas	3.0 l/min
Scan End	1000 m/z	Set Charging Voltage	2000 V	Set Divert Valve	Source
		Set Corona	0 nA	Set APCI Heater	0 °C

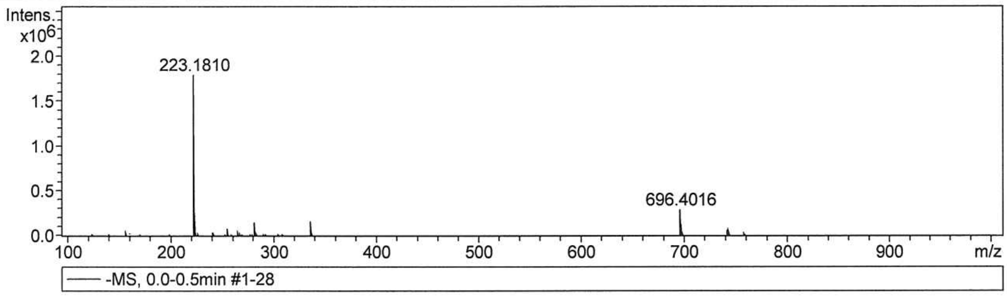

#	m/z	Res.	S/N	I	I %	FWHM
1	223.1810	26744	75118.6	1788977	100.0	0.0083
2	696.4016	21244	12079.6	293429	16.4	0.0328

Compound 7B: 30-(3-((dimethoxyphosphoryl)amino)prop-1-en-2-yl)-28-*O*-but-2-ynoyl botulin

Figure S23. ^{1}H NMR, compound **7B**

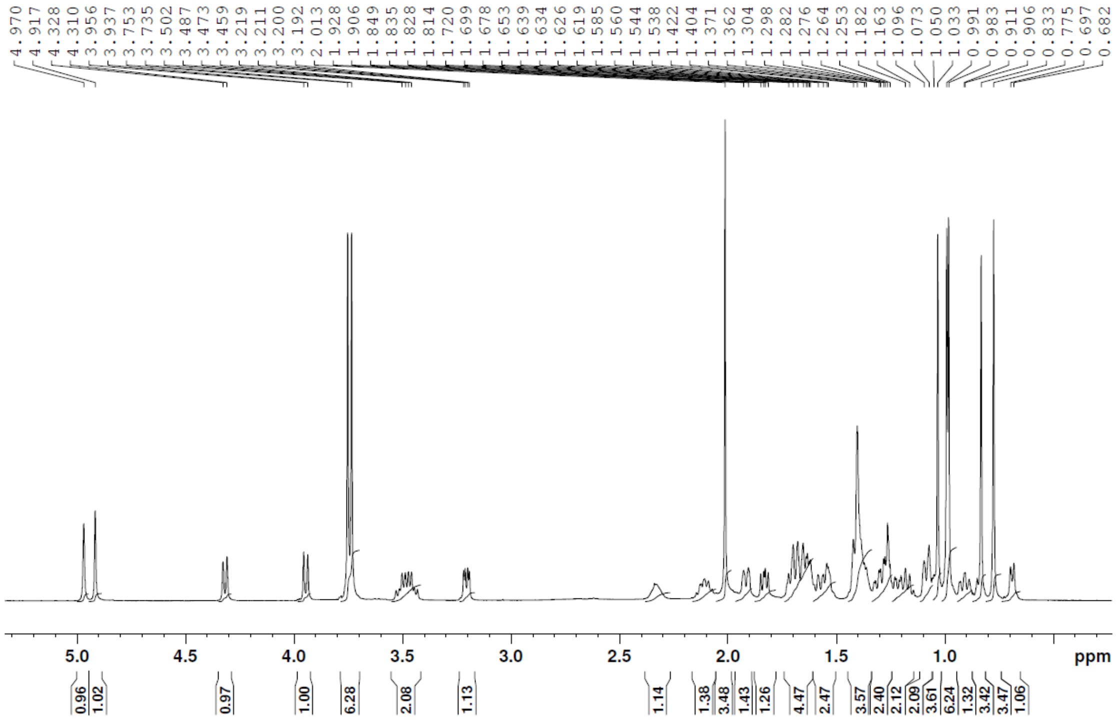

Figure S24. ^{13}C NMR, compound **7B**

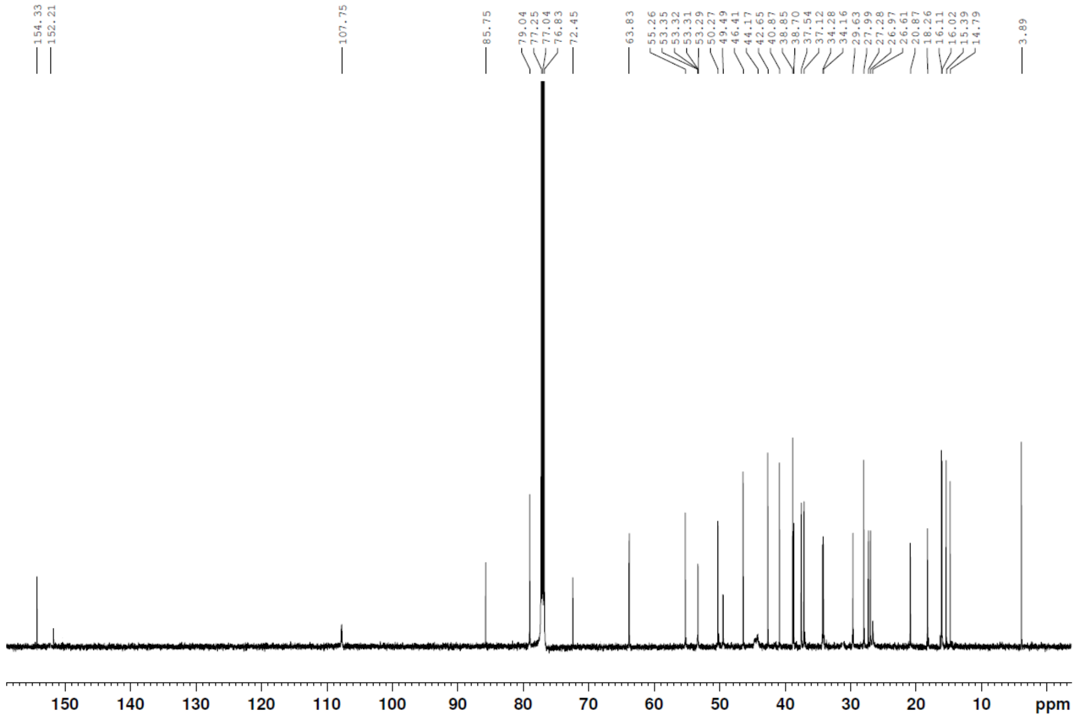

Figure S25. ^{31}P NMR, compound **7B**

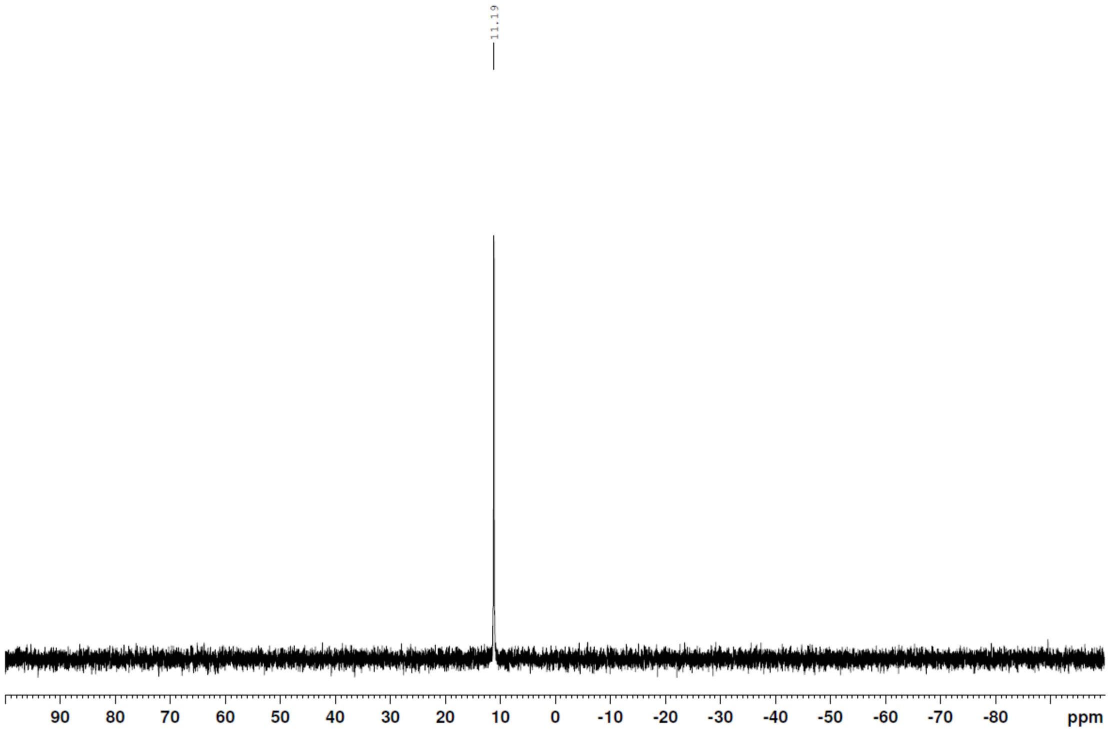

Figure S26. HRMS, compound **7B**

Analysis Name	D:\Data\specs\PM_20251103\performance 2\ECH 292B.d			
Method	low_mass_positive.m	Operator	KM	
Sample Name	Tune_neg_high_post	Instrument	impact II	1825265.10082
Comment				

Acquisition Parameter

Source Type	ESI	Ion Polarity	Negative	Set Nebulizer	0.3 Bar
Focus	Active	Set Capillary	2500 V	Set Dry Heater	200 °C
Scan Begin	100 m/z	Set End Plate Offset	-500 V	Set Dry Gas	3.0 l/min
Scan End	1000 m/z	Set Charging Voltage	2000 V	Set Divert Valve	Source
		Set Corona	0 nA	Set APCI Heater	0 °C

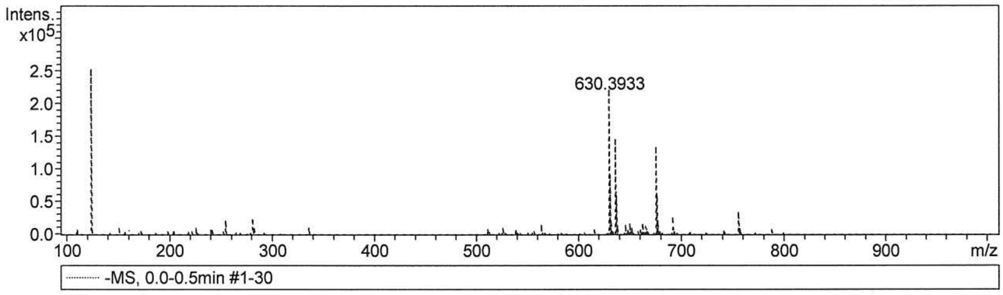

#	m/z	Res.	S/N	I	I %	FWHM
1	630.3933	20827	6313.8	215617	100.0	0.0303
2	636.3676	20266	4276.4	147605	68.5	0.0314
3	676.3986	20025	3636.3	127916	59.3	0.0338

Compound 8: 30-(3-((dimethoxyphosphoryl)amino)prop-1-en-2-yl)betulonic acid

Figure S27. ^{1}H NMR, compound **8**

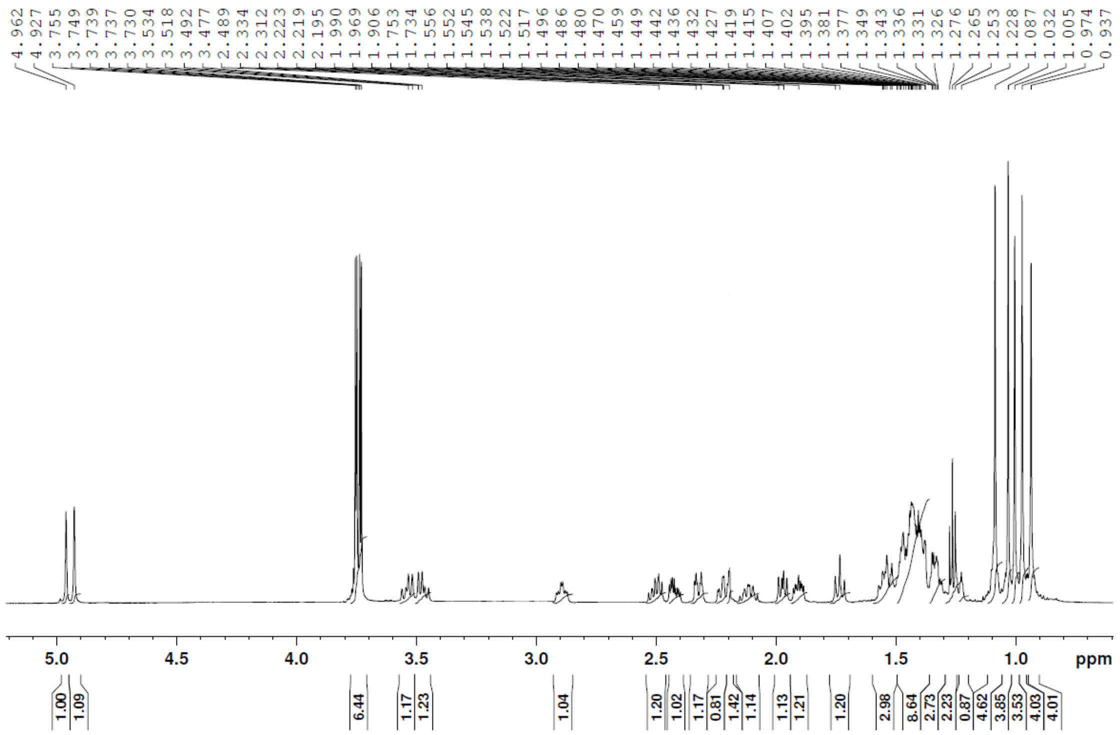

Figure S28. ^{13}C NMR, compound **8**

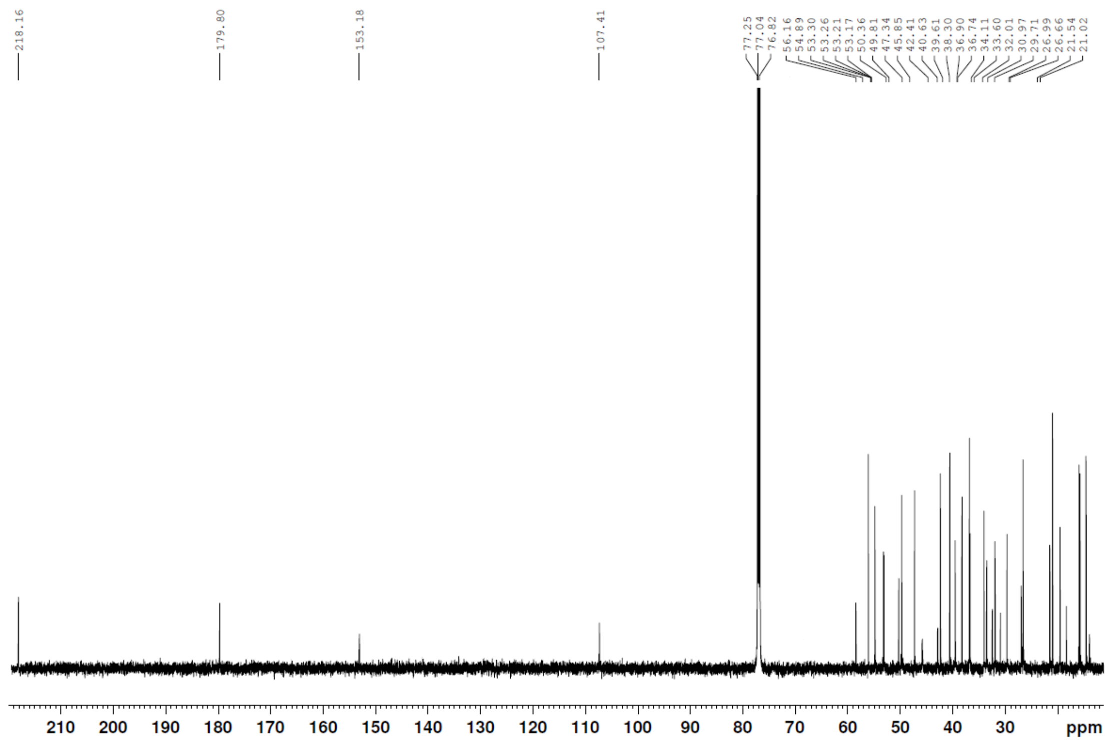

Figure S29. ^{31}P NMR, compound **8**

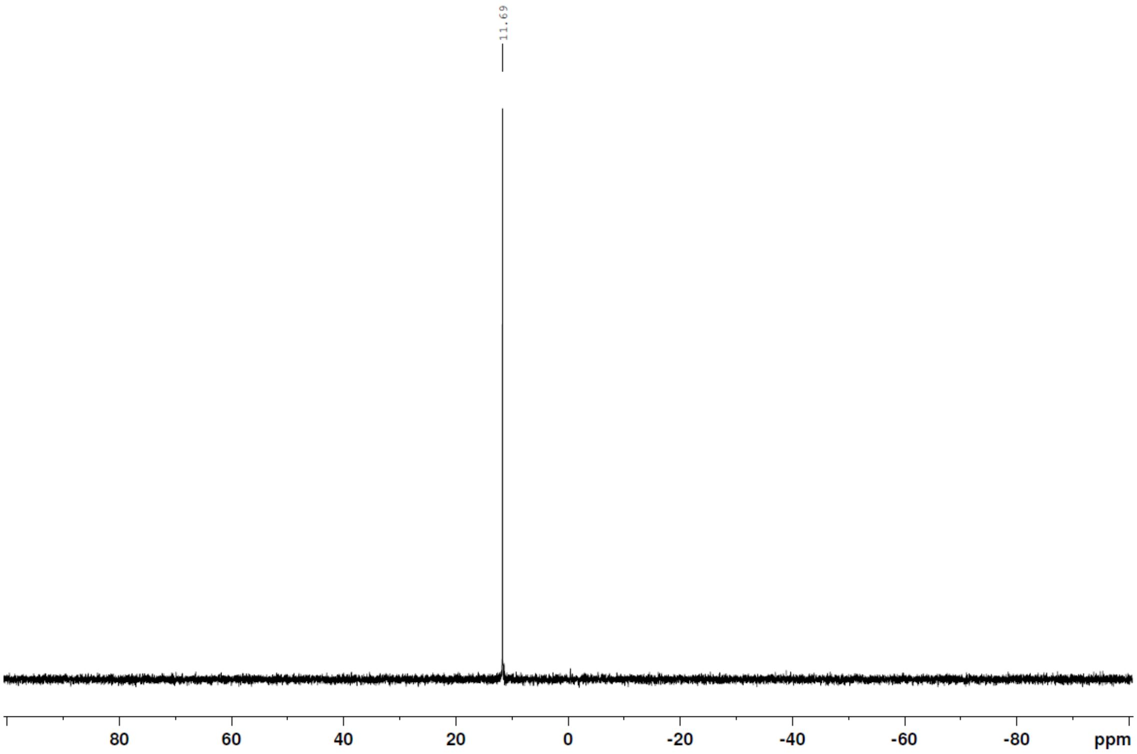

Figure S30. HRMS, compound **8**

Analysis Name	D:\Data\ECh234.d			
Method	low_mass.m	Operator	KM	
Sample Name	TM Low concentration	Instrument	impact II	1825265.10082
Comment				

Acquisition Parameter

Source Type	APCI	Ion Polarity	Negative	Set Nebulizer	2.0 Bar
Focus	Active	Set Capillary	4000 V	Set Dry Heater	200 °C
Scan Begin	100 m/z	Set End Plate Offset	-500 V	Set Dry Gas	5.0 l/min
Scan End	1000 m/z	Set Charging Voltage	2000 V	Set Divert Valve	Source
		Set Corona	2000 nA	Set APCI Heater	450 °C

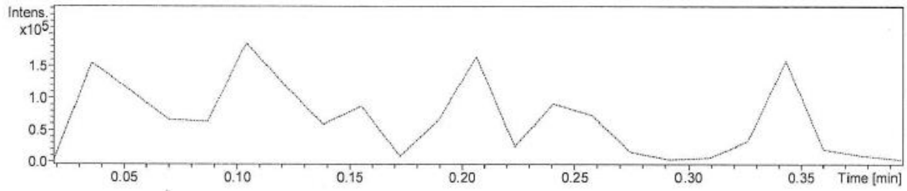

-MS, 0.0-0.4min #1-23

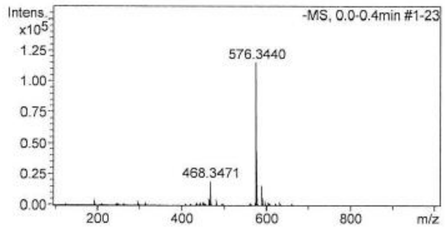

#	m/z	Res.	S/N	I	I %	FWHM
1	468.3471	36306	1416.2	19865	17.2	0.0129
2	576.3440	44749	7640.1	115191	100.0	0.0129
3	590.3235	39230	1051.9	15868	13.8	0.0150

Compound 9: 30-(3-((dimethoxyphosphoryl)amino)prop-1-en-2-yl)betulinic acid

Figure S31. ^{1}H NMR, compound **9**

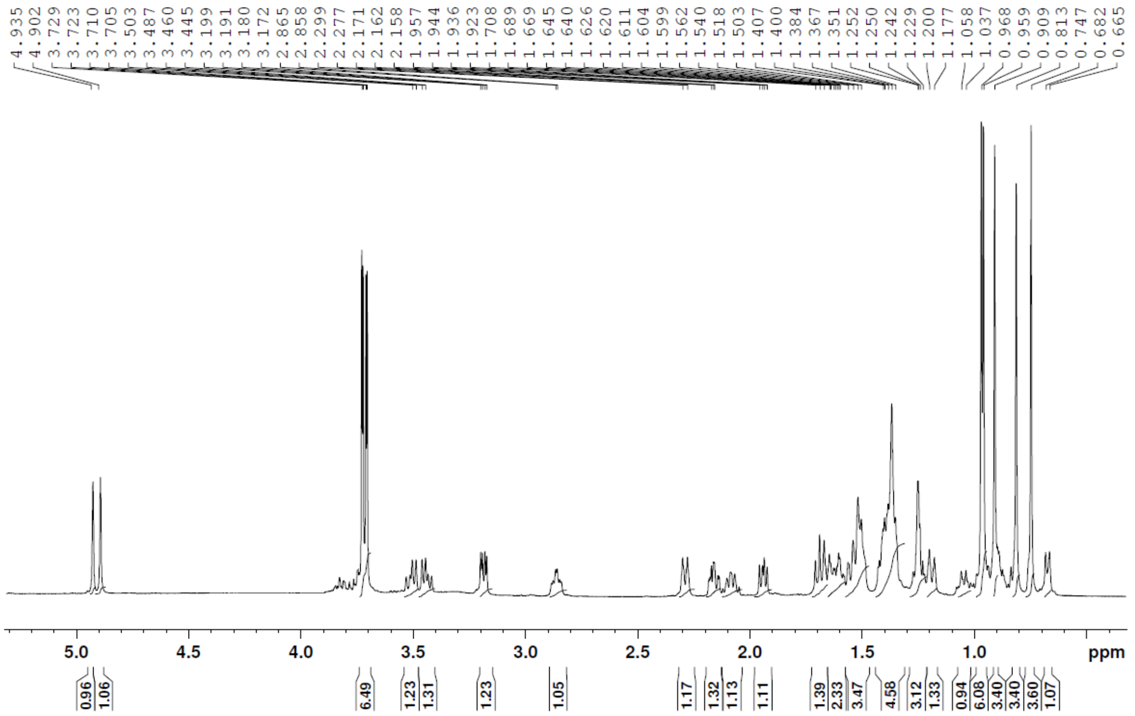

Figure S32. ^{13}C NMR, compound **9**

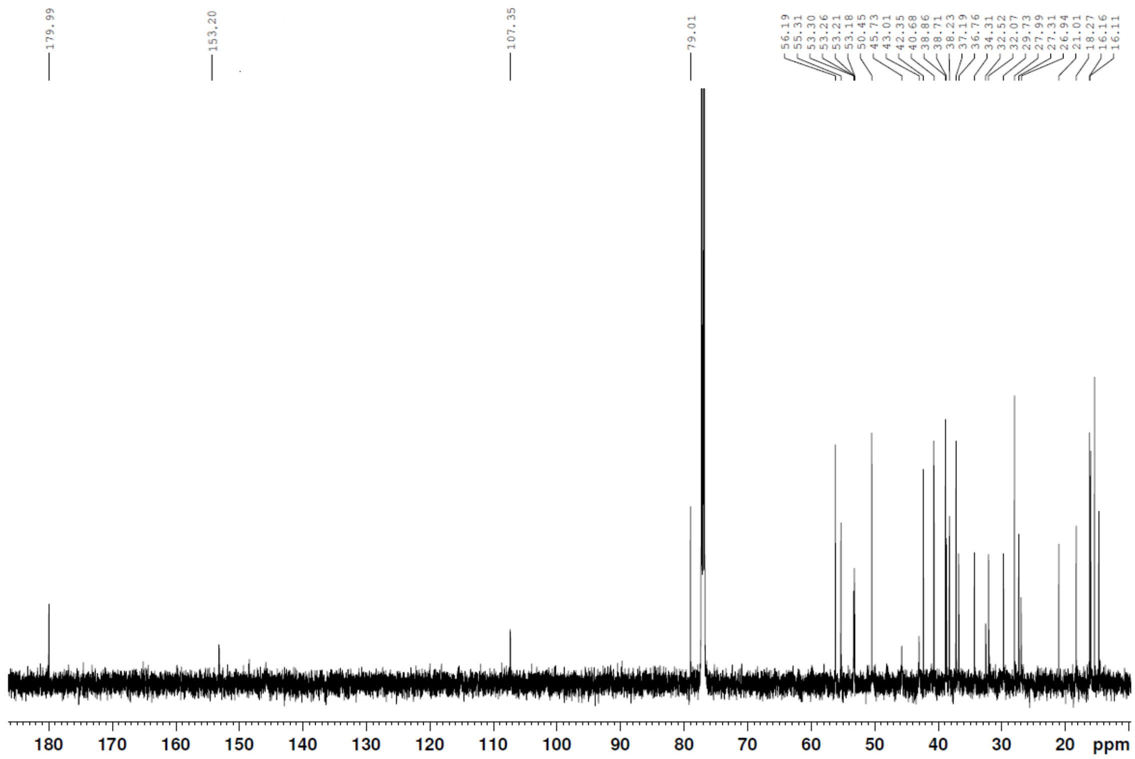

Figure S33. ^{31}P NMR, compound **9**

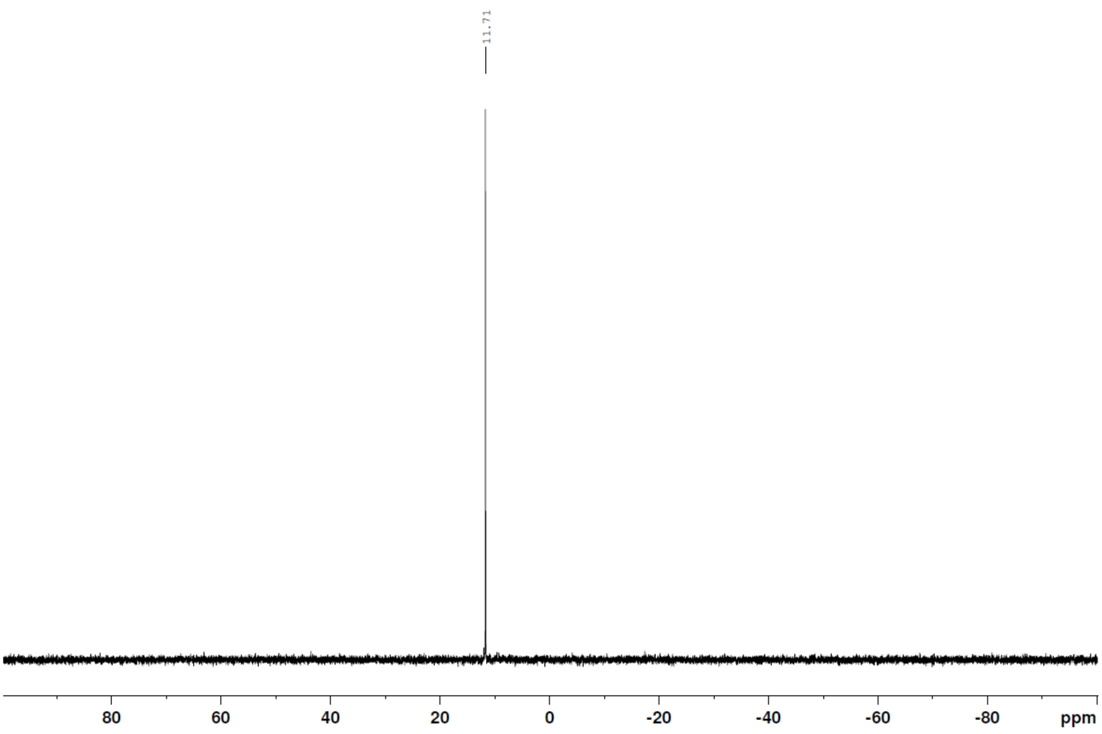

Figure S34. HRMS, compound **9**

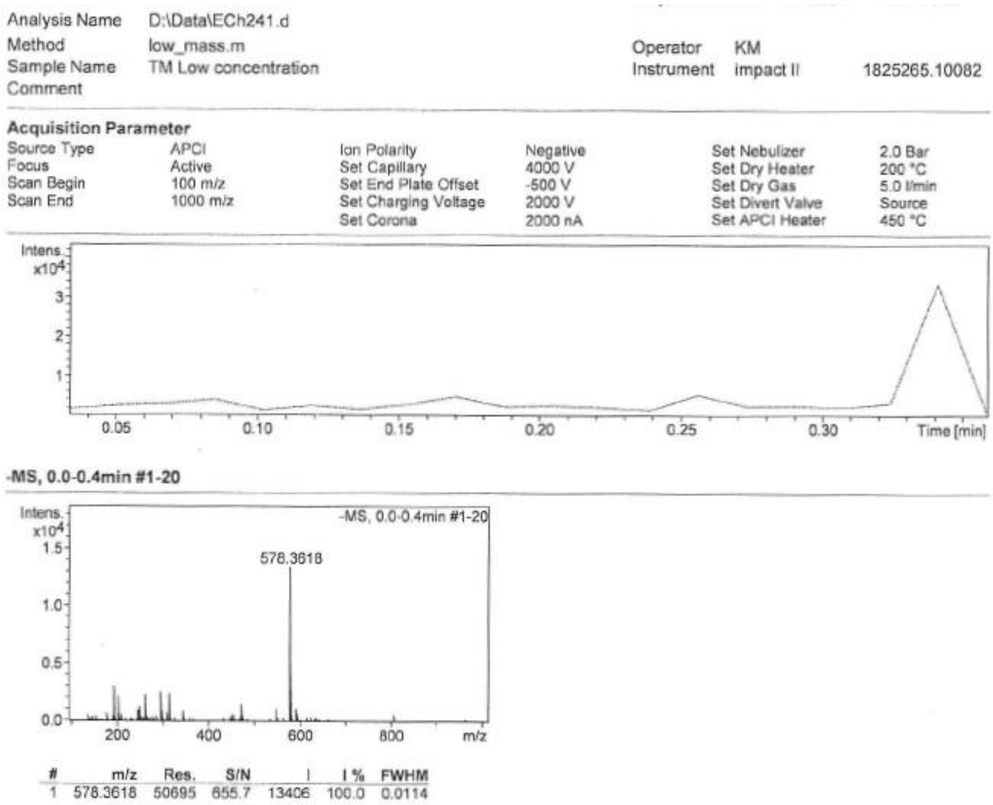

Table S1. Lipophilicity parameters of standard compounds; determined experimentally (RM0; mobile phase acetone:buffer Tris, pH 7.4) and literature values ($logP_{lit}$)

**Reference compound**	**R_{M0}**	**log P_{lit}**	**log P_{TLC}**	***b***	***r***
acetanilide	0.75	1.21	1.35	-0.016	0.963
prednisone	1.04	1.62	1.68	-0.021	0.943
4-bromoacetophenone	2.03	2.43	2.81	-0.027	0.993
benzophenone	2.72	3.18	3.60	-0.035	0.977
testosterone	1.87	3.32	2.63	-0.026	0.985
anthracene	3.48	4.45	4.47	-0.042	0.999
9-phenylanthracene	3.94	6.01	5.00	-0.047	0.997
dibenzyl	4.23	4.79	5.33	-0.049	0.998
DDT[a]	5.05	6.38	6.27	-0.059	0.997
cholesterol	7.91	8.74	9.54	-0.075	0.997

[a]DDT- dichlorodiphenyltrichloroethane

b is the slope and *r* is the correlation coefficient for the linear relationship $R_M = R_{M0} + bC$

The below calibration curve equation was used to determine the experimental lipophilicity values ($logP_{TLC}$) of the tested compounds:

$logP_{TLC} = 1.1442\ R_{M0} + 0.4921$ ($r = 0.986$, SD=0.513)
